# A Conserved, Serine-Rich Protein Plays Opposite Roles in N-Mediated Immunity against TMV and N-Triggered Cell Death

**DOI:** 10.3390/v15010026

**Published:** 2022-12-21

**Authors:** Qingling Zhang, Jubin Wang, Xi Zhang, Yingtian Deng, Feng Li

**Affiliations:** Key Laboratory of Horticultural Plant Biology (MOE), College of Horticulture and Forestry Sciences, Huazhong Agricultural University, Wuhan 430070, China

**Keywords:** SR proteins, plant immunity, *N* gene, tobacco mosaic virus, cell death, RNA silencing

## Abstract

Plant nucleotide-binding, leucine-rich, repeat-containing proteins (NLRs) play important roles in plant immunity. NLR expression and function are tightly regulated by multiple mechanisms. In this study, a conserved serine/arginine-rich protein (SR protein) was identified through the yeast one-hybrid screening of a tobacco cDNA library using DNA fragments from the *N* gene, an NLR that confers immunity to tobacco mosaic virus (TMV). This SR protein showed an interaction with a 3′ genomic regulatory sequence (GRS) and has a potential role in regulating the alternative splicing of *N*. Thus, it was named SR regulator for *N*, abbreviated SR4N. Further study showed that SR4N plays a positive role in *N*-mediated cell death but a negative role in *N* protein accumulation. SR4N also promotes multiple virus replications in co-expression experiments, and this enhancement may not function through RNA silencing suppression, as it did not enhance 35S-GFP expression in co-infiltration experiments. Bioinformatic and molecular studies revealed that SR4N belongs to the SR2Z subtype of the SR protein family, which was conserved in both dicots and monocots, and its roles in repressing viral immunity and triggering cell death were also conserved. Our study revealed new roles for SR2Z family proteins in plant immunity against viruses.

## 1. Introduction

Plant immunity against viruses consists of multiple mechanisms. Plant Dicer-like (DCL) enzymes cut viral double-stranded RNA into 21- to 24-nucleotide (nt) small interfering RNAs (siRNA) to activate siRNA-based antiviral immunity. These virus-derived siRNAs (vsiRNAs) are loaded into Argonaute (AGO) proteins to form an RNA-induced silencing complex (RISC) with one strand retained. Then, vsiRNAs guide RISCs to silence viruses based on the complementarity between vsiRNA and viral RNA [1,2]. RISCs that contain AGO1 or AGO2 and are programed by DCL4-dependent 21 nt vsiRNA and DCL2-dependent 22 nt visRNA usually trigger viral RNA cleavage and translation inhibition, thus providing immunity against both RNA and DNA viruses at the post-transcriptional level. In contrast, RISCs containing AGO4 and DCL3-dependent 24 nt vsiRNAs suppress DNA viruses via transcriptional silencing [3,4]. The plant RNA-dependent RNA Polymerase 1 (RDR1) and RDR6 participate in this antiviral immunity by amplifying the production of vsiRNAs to potentiate this pathway [5]. siRNA-based immunity is successful in defending plants from most virus infections; however, some viruses encode various suppressors of RNA silencing (VSRs) that counteract siRNA pathways with diverse mechanisms to allow viruses to thrive in host plants [6]. In plant-virus coevolution, plants evolved nucleotide-binding, leucine-rich, repeat-containing protein (NLR)-based immunity that triggers rapid defense reactions to contain pathogens at the initial infection site, usually accompanied by programmed cell death upon the recognition of viral effectors by cognate NLRs. As NLRs provide race-specific immunity against viruses and other types of pathogens and pests, their effectors evolve very fast; plant NLRs had gone through extensive duplications and diversification and formed a large gene family during their co-evolution with microbes [7]. The *N* gene that confers immunity against tobacco mosaic virus (TMV) was the first NLR to recognize plant viruses and is defined as one of the major types of NLR which has the N-terminal Toll/interleukin-1 receptor-like (TIR) domain and is called TNL [8]. Recently, many excellent studies had elucidated the mechanisms of NLR-mediated defense signaling. TNL proteins undergo oligomerization upon recognizing pathogen effectors and catalyze the synthesis of several nucleotide-derived small signaling molecules. These molecules further trigger the formation of heterodimers between enhanced disease susceptibility 1 (EDS1) and its paralogs, which, in turn, activate helper NLR to form a calcium channel on the cell membrane and trigger calcium influx and, eventually, cell death [9,10,11].

Although NLRs confer immunity against devastating pathogens and are important for plant success in evolution, there is a fitness cost for plants carrying NLRs in their genomes, as their expression is harmful to plant growth and reproduction in the absence of cognate pathogens [12]. To counteract the fitness cost associated with NLRs, plants evolved multilayer mechanisms to suppress NLR expressions and functions during normal growth.

In the *Solanaceae* family, many NLR genes have been found colocalized with various transposable elements, which generate abundant 24 nt siRNAs that may transcriptionally silence nearby NLR genes through the RNA-directed DNA methylation (RdDM) pathway [13]. Recently, whole-genome cytosine methylation analysis in the common bean revealed that among 90 NLRs targeted by 24 nt siRNA, 90% of them were methylated [14]. Consistent with the negative role of DNA methylation in NLR regulation, demethylation due to the DEMETER enzyme is required to enhance TNL expression and resistance to bacterial and fungal infections [15]. Thus, RdDM functions at the initial stage of NLR expression by repressing its transcription.

At the post-transcriptional level, numerous miRNAs have been identified targeting a major portion of the NLR transcripts in various plant species, and many of these NLR-targeting miRNA genes evolved through the inverted duplication of NLR gene fragments; for example, the *Solanaceae*-specific MIR6019/6020 family was derived from the inverted duplication of TIR-coding sequences from *N* gene ancestors [12,16,17]. Some of these miRNAs were 22 nt in length and triggered phased siRNA synthesis from targeted NLR mRNAs or NLR-derived noncoding transcripts to broaden their NLR silencing targets [16,18,19]. At the post-translational level, the ubiquitin-proteasome system (UPS) plays an important role in regulating NLR protein levels [20]. In Arabidopsis, mutations in the UPS pathway components, *CPR1*, *MUSE3*, and *AtCDC48A*, resulted in the overaccumulation of the TNL SNC1 protein and an enhanced disease resistance phenotype or autoimmunity [20]. Tobacco UBR7, a portative E3 ligase of the UPS, has been reported to downregulate the N protein level by binding to its TIR domain. Interestingly, the presence of p50 from TMV inhibited UBR7-TIR interactions, which provided a mechanism for plants to activate N upon infection [21].

Besides the abovementioned regulatory mechanisms, many NLRs are also subjected to alternative splicing (AS), and many NLRs require multiple isoforms of transcripts to confer full resistance [22]. For example, full resistance to TMV conferred by the *N* gene requires full-length N and truncated N (N^tr^) proteins that are translated from N_S_ and N_L_, respectively, which contain an alternatively spliced exon (AE) encoding a premature stop codon [23]. Mechanisms of AS regulation are well-studied in animal systems and serine/arginine-rich proteins have been shown to play important roles in regulating AS events [24]. However, it is not well understood how the NLR AS is regulated and what the role of plant SR in plant immunity against viruses is.

In this study, we identified a tobacco serine/arginine-rich (SR) protein in a yeast one-hybrid (Y1H) screening of a tobacco cDNA library using *N* gene DNA fragments as bait. This SR protein interacts with *N* gene terminator sequences. It significantly reduced overall N/N^tr^ protein levels when co-expressed with *N*, and it has a moderate effect on the *N_S_*/*N_L_* transcript ratio, suggesting it may also affect the AS of N transcripts. Thus, we named this protein SR regulator for the *N* gene, abbreviated as SR4N. The overexpression of *SR4N* promoted TMV replication in both wild-type and *N*-transgenic plants, suggesting it is a negative regulator of plant immunity. However, knocking-down *SR4N* via virus-induced gene silencing (VIGS) inhibited N-p50-triggered cell death, and the overexpression of *SR4N* alone was sufficient to trigger cell death, suggesting *SR4N* is a positive regulator of cell death. Bioinformatics analysis showed that SR4N belongs to a conserved SR2Z subfamily of SR proteins. *SR4N* homologs in tomato, Arabidopsis, and rice also play negative roles in plant immunity against TMV and N protein accumulation and a positive role in cell death. Our results uncovered the opposite roles of RS2Z-type SR proteins: repressing plant immunity against viruses and promoting cell death.

## 2. Materials and Methods

### 2.1. Plant Materials and Virus Strains

Wild-type *N. benthamiana* was used for transient expression assay and VIGS assay. *N. tabacum* (SR1), *S. lycopersicum* (A57), *A. thaliana* (Col0), and *Oryza sativa* L. leaves were used to clone the SR4N gene. TRV2-GFP [25], TMV-GFP [16], TBSV-GFP [26], and pMS4 [26] vectors used for VIGS assay and resistance assay were described earlier. *N. tabacum* transgenic line TG34 was used for SR4N expression analysis, which was transformed with the tobacco mosaic virus resistance gene *N* and described earlier [27].

### 2.2. Construction of SR4N Expression, VIGS, and Yeast One-Hybrid Vector

Total RNA was extracted from the leaves of tobacco/tomato/Arabidopsis/rice/*N. benthamiana*. After reverse transcription using M-MLV Reverse Transcriptase (Thermo Fisher Scientific, Waltham, MA, USA), the tobacco, tomato, Arabidopsis, and rice SR4N coding sequences were amplified and cloned into plant expression vector pH7LIC8.1 digested by *Stu*I (NEB, Ipswich, MA, USA) through homologous recombination, which expressed the SR4N protein with N-terminal 3 × HA tags (Supplementary Data S1).

For VIGS experiments, about 200bp of SR4N gene fragments was amplified from N. benthamiana cDNA and cloned into TRV2-GFP expression vector digested by SmaI (NEB, Ipswich, MA, USA) through homologous recombination.

The N promoter containing 5′-UTR was divided into 11 fragments (NP1 to NP11), N intron III was divided into 5 fragments (NI1 to NI5), and N 3′-GS terminator containing 3′-UTR was divided into 3 fragments (NT1 to NT3). These fragments were amplificated from SPDK450 vector and cloned into pABAi vector digested by *Sac*I (NEB, Ipswich, MA, USA) and *Xho*I (NEB, Ipswich, MA, USA) through T4 DNA ligase. The SR4N were amplified from pH7LIC8.1-SR4N vector and cloned into yeast expression vector pGADT7LIC2.0 via homologous recombination.

Primers used for constructing these vectors are listed in Supplementary Table S1.

### 2.3. Construction of Tobacco cDNA Library

Total RNA was extracted from leaves collected from 1-, 3-, and 6-week-old tobacco plants and used for the construction of a prey cDNA library using Matchmaker Gold Yeast One-Hybrid Library Screening System Kit (Clontech, Mountain View, CA, USA), according to the manufacturer’s instructions.

### 2.4. Yeast One-Hybrid Screening

Nineteen bait vectors containing different *N* gene fragments (NPn, NTn, NIn, etc) were individually transformed into yeast strain Y1HGold. The bait strain was grown on yeast nitrogen base (YNB) plates with different ABA concentrations, and 400 ng/mL was found to provide optimal selection pressure. Before mating, the fourteen bait yeast colonies, except for NT1, NI5, NP1, NP17, and NP19 bait yeast with autoactivation, were picked, resuspended in YNB liquid medium to OD600 of 0.5, 100 μL of each strain was mixed, and 50 mL of fresh YNB culture was seeded into a 250 mL flask. The mixed bait culture was grown at 28 °C for 16–20 h until OD600 reached 0.8; then, it was spun down and resuspended in 4–5 mL YNB medium. A tobacco cDNA library yeast culture of 1 mL was added to 45 mL 2× YPDA medium together with bait culture in a 2 L flask for 20–24 h while gently shaking at 30–50 rpm. After mating, the yeast culture was spun down, washed three times with 50 mL 2× YPDA, and resuspended in 10 mL 2× YPDA. Finally, the yeast culture was plated on YNB media (with 1000 ng/mL ABA, 2% Glucose, 1×-URA) and cultivated at 30 °C for 3–5 days. The well-grown colonies were picked up and subjected to a colony PCR using primer pair ZH1010/1011 (see Table S1). PCR products longer than 250 nt were chosen for sequencing analysis.

### 2.5. VIGS and Overexpression in N. benthamiana

To knockdown *SR4N* with virus-induced gene silencing, about 200 bp of cDNA fragments from NtaSR4Na and NtaSR4Nb was cloned into the TRV2-GFP vector to produce TRV2-GFP-NtaSR4Na and TRV2-GFP-NtaSR4Nb. Each of these TRV2 agrobacteria, including a TRV2-GFP control agrobacterium, was co-infiltrated into the leaves of 3-week-old *N. benthamiana* plants with TRV1 agrobacterium at a final OD600 of 0.1 for each strain. Thirty plants were used for each treatment, and experiments were repeated three times.

For the overexpression of *SR4N* to induce cell death, agrobacteria at an OD600 of 0.5 were used, while for co-infiltration with N and TMV-GFP, agrobacteria at an OD600 of 0.1 were used. Agrobacterium cultivation and preparation for infiltration were conducted as described earlier [28] with the following modifications: the agrobacteria were resuspended in 10 mM MgCl_2_ and 250 µM acetosyringone to various OD600s before infiltration. Five plants were used for co-infiltration, and experiments were repeated three times.

### 2.6. Expression Analysis of SR4N

To detect SR4N expression in different timepoints after TMV infection, 6-week-old TG34 leaves were infected with TMV-U1, total RNA was extracted from the infected leaves at a different timepoint, and 2 µg total RNA after genomic DNA digesting by DNaseI (Transgen, Beijing, China) was used to obtain plant cDNA using oligo-dT and M-MLV reverse transcription enzyme (Thermo Fisher Scientific, Waltham, MA, USA). The *NtaSR4N* and *NtaTubulin* genes were amplified for 35 and 28 cycles in PCR, respectively. The gene expression value was calculated using ImageJ 1.50i software (NIH, Bethesda, MD, USA). Primers are listed in Supplementary Table S1.

RNA-seq data from tobacco and tomato leaf samples from unpublished laboratory data were used. High-throughput RNA sequencing data from tobacco, tomato, Arabidopsis, and rice tissue samples were downloaded from NCBI (Accession numbers: tobacco root SRR19895110, tobacco flower SRR19895113, tobacco seeds SRR13908972, *N. benthamiana* seed SRR21782212, *N. benthamiana* flower SRR696938, *N. benthamiana* leaf SRR696940, *N. benthamiana* root SRR696961, tomato root SRR19075381, tomato flower SRR14102833, tomato seeds SRR13787019, Arabidopsis root SRR656215, Arabidopsis flower SRR656217, Arabidopsis seeds ERR6351727, Arabidopsis leaf SRR656216, rice root SRR8051560, rice flower SRR8051546, rice seeds SRR7974062, rice leaf SRR8051557). The NGS transcriptome data were filtered and trimmed using Trimmomatic [29] and then aligned to the tobacco, tomato, Arabidopsis, and rice genomes using the hisat2 software with the default settings [30]. The alignment results were sorted using samtools [31]. The normalized gene expression profile was calculated using the cuffquant and cuffnorm (—library-norm-method classic-fpkm) program from the cufflinks package [32]. The *SR4N* gene expressions from different species were extracted from a merged expression matrix. After normalizing using log 10, a heatmap of gene expressions was drawn using the R package pheatmap (scale = row).

### 2.7. Protein Extraction and Western Blot Analysis

To prepare the total protein sample for Western blot analysis, 0.1 g of leaf tissue and a steel bead was placed in a 2 mL microcentrifuge tube (MCT). The MCT was placed in a Qiagen TissueLyser II (Qiagen, Hilden, Germany) and ground for 15 s at 30 rpm. Then, 200 μL of extraction buffer (150 mM NaCl, 20 mM Tris-HCl pH7.5, 1 mM EDTA, 0.1% Triton X-100, 10% glycerol, 5 mM DTT, 2 mM NaF, 1 mM PMSF, and 1× protease inhibitor) was added and mixed well. The MCT was then subjected to centrifugation at 12,000 rpm for 10 min at 4 °C. The upper clear lysate was kept at 4 ℃ for immediate use or kept at −80 °C for long-term storage.

The TGX stain-free FastCast Acrylamide Kit (Bio-Rad, Hercules, CA, USA) was used to prepare SDS PAGE gel for protein separation. Before loading, the prepared total protein sample was mixed with an equal volume of 2× SDS sample buffer (0.125 M Tris-HCl (pH 6.8), 4% (*w*/*v*) SDS, 20% (*v*/*v*) glycerol, 0.3 M β–thio-ethanol, 0.05% (*w*/*v*) Bromophenoblue), heated in 100 °C water bath for 5 min, and 20 μL of each was loaded onto the gel, together with a protein marker (Thermo Scientific, Waltham, MA, USA). The gel was first run at a constant voltage of 80 V for 30 min until the protein sample ran into the resolving gel and then ran at a constant 120 V for 45 min. N and actin were detected on one membrane via sequential hybridization using different antibodies and stripping. N^tr^ was detected on another membrane run at the same time.

Protein was transferred from the gel to the PVDF membrane (GE Healthcare, Boston, MA, USA) using Trans-Blot Turbo (Bio-Rad, Hercules, CA, USA) according to the manufacture’s protocol. The membrane was then blocked in 1 × TBS buffer with 5% fat-free milk in a horizontal shaker (Orbital Shaker TS-2, Meditry Instrument Co., Ltd., Jiangyin, China) for 1–2 h, agitated at 100 rpm. Primary antibodies were added with the following dilution times: 1:5000 for anti-FLAG (HT201, Transgen, Beijing, China), anti-MYC (M20002, Abmart, Shanghai, China), anti-HA (M20003, Abmart, Shanghai, China), and anti-actin (AC009, ABclonal, Wuhan, China). They were incubated for 2 h. Next, buffer with primary antibody was removed, and the membrane was washed thrice with 1× TBST buffer, 10 min each time. Secondary antibody HRP-conjugated Affinipure Goat Anti-Mouse IgG (H+L) (66002-1-Ig, Proteintech, Chicago, IL, USA) with 1:5000 dilution was added and incubated for 1 h. Finally, the membrane was washed three times with 1× TBST again and developed using Clarity Western ECL Substrate (Bio-Rad, Hercules, CA, USA) and imaged using ChemiDoc XRS + (Bio-Rad, Hercules, CA, USA) according to the manufacturer’s instructions.

### 2.8. Phylogeny Analysis of SR Family Proteins

Arabidopsis and rice SR protein names were downloaded from a previous report [31], and SR protein sequences were extracted from whole-protein databases (Supplementary Data S2). Tomato, tobacco, and *N. benthamiana* SR protein sequences were identified from protein databases [33] through the blastp program in the ncbi-blast software (the minimum score was 100) and hmmer software, which recognizes RNA recognition motifs (Pfam: PF00076.23) [34,35] (Supplementary Data S2). The SR protein sequences from these four species were integrated together to obtain a multiple sequence alignment result using a clustalW command with default settings in MEGA11 and constructing a phylogenetic tree using the maximum likelihood method in MEGA11 [36]. The final phylogenetic tree picture was displayed by the R package ggtree [37].

## 3. Results

### 3.1. Identification of SR4N in Y1H Screen

**Figure 1:**
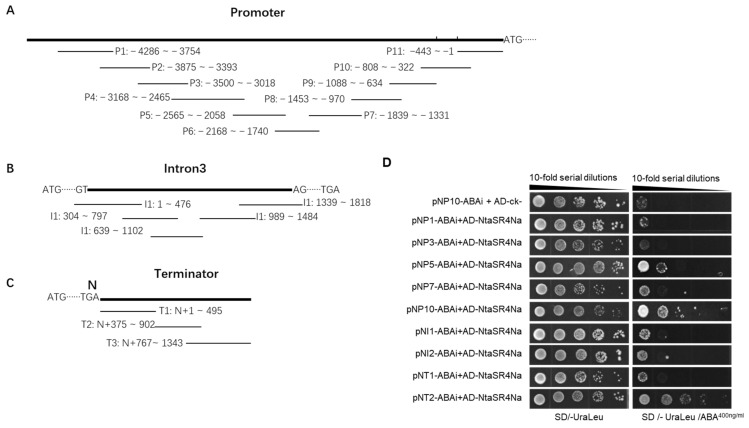
Interaction between NtaSR4N and the *N* gene in a yeast one-hybrid assay. (**A**–**C**) Diagrams of *N* promoter, Intron 3, and terminator DNA fragments used in bait vectors. (**D**) Point-to-point Y1H tests. Bait and prey vectors in each yeast strain are indicated to the left. Medium composition is indicated at the bottom.

To identify potential transcriptional regulators, we conducted a yeast one-hybrid screening using a homemade tobacco cDNA library and a mixture of around 500 bp overlapping DNA fragments from the *N* gene promoter, AE-containing intron, and terminator (Figure 1A–C). In total, 185 clones were picked up from the selection plates and 39 clones with cDNA insertions were identified (Figure S1A). The PCR products were gel-purified and sent for Sanger sequencing. Sequence annotation identified 17 cDNA with known protein functions, and 1 of them was annotated as being serine/arginine-rich splicing factor RS2Z33-like (Figure S1B). As *N* is alternatively spliced, and its AS is important for its function, this potential splicing factor for the *N* gene (abbreviated as SR4N) caught our attention and was further tested in a point-to-point Y1H assay with each *N* gene fragment. The results showed that yeast containing SR4N and each prey DNA fragment grew well on the medium without Ura and Leu; similar results were observed for yeast containing the corresponding prey DNA and negative control bait, indicating all yeast strains are active under nonselective conditions (Figure S1C). On the selection medium with ABA, yeast containing the NT2 fragment showed the best growth; yeast with NI1, NI2, and NP10 showed moderate growth; yeast with NP2 and 5 showed minimum growth; and all the other yeast strains did not grow (Figure S1C). To further test the potential interactions between these DNA fragments with SR4N, the yeast strains showing growth were further tested in a dilution assay. A ten-fold series dilution was made for each strain and spotted on both the nonselective and selective media. All strains grew on the nonselective medium at 10^4^ × dilution, while only yeast strains containing NT2 and NP10 grew at 10^3^ × and 10^2^ × dilution, respectively, on the selective medium (Figure 1D). These results suggested that SR4N had a moderate interaction with the NT2 and NP10 fragments of *N*.

### 3.2. SR4N Expression Is Stimulated by TMV–N Interaction and Developmentally Regulated

**Figure 2:**
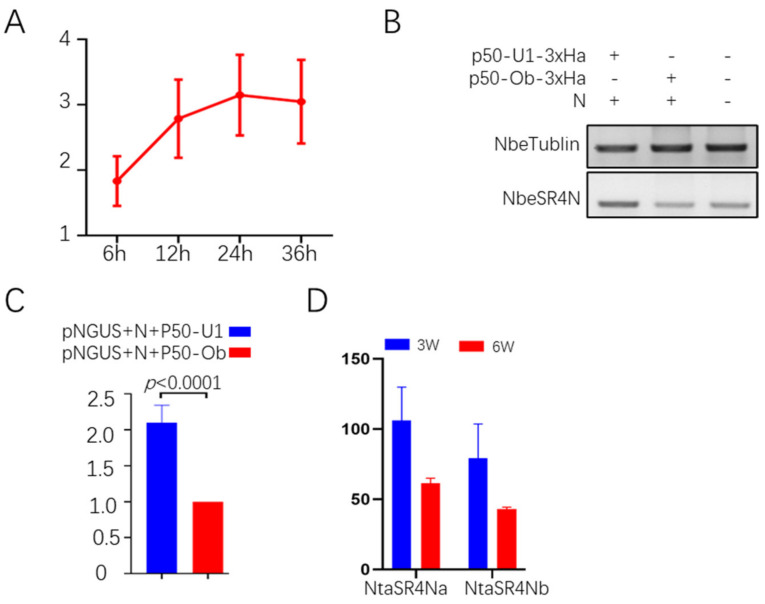
Expression pattern of NtaSR4N. (**A**) Quantitative RT-PCR analysis of the NtaSR4N level during TMV infection in *N* plants. The Y-axis is an arbitrary unit of relative expression levels normalized to NtaTubulin. The X-axis is the number of hours after inoculation. (**B**) Detection of NbeSR4N expression via semiquantitative RT-PCR in transient assays. Combinations of vectors co-infiltrated are indicated on top of each lane. (**C**) Quantitation of DNA bands in (**B**) using ImageJ. A two-tailed Student’s *t*-test was used for comparison between the two samples. GraphPad Prism 8 was used for statistical analyses. (**D**) Expression levels of NtaSR4Na and b in 3- and 6-week-old plant leaves. Y-axis is the number of mRNA-seq reads per kb per million total reads (RPKM). Expression data represent mean values for three biological replicates (*n* = 3).

To test the relevance of SR4N with N-mediated TMV immunity, we set out to investigate its response during a TMV infection in the N-transgenic plant TG34. Total RNA samples were harvested from inoculated leaves at four different timepoints, 6, 12, 24, and 36 h post-inoculation (HPI). Semi-quantitative RT-PCR was conducted to analyze the expression levels of NtaSR4N in different samples, and the results showed that the SR4N expression level clearly increased from 6 to 12 HPI (Figure 2A), indicating its expression is stimulated by N–TMV interaction as N recognizes TMV through its p50 region [38]. We further tested whether the p50-N interaction stimulates SR4N expression. For this, p50 from the TMV U1 strain—which is recognized by N—and Ob strain—which is not recognized by N—were co-expressed in *N. benthamiana* leaves via agroinfiltration. Semi-quantitative RT-PCR using NbeSR4N (*N. benthamiana* SR4N) primers resulted in a clearly stronger SR4N DNA band in the p50-U1 and N-co-infiltrated sample than in the p50-Ob and N-co-infiltrated sample or the un-infiltrated control sample, while the tubulin bands were of similar intensity in all samples (Figure 2B). An ImageJ analysis of the gel results showed that the levels of SR4N in the p50-U1-N-expressed sample was about 2.1-fold of that in the p50-Ob-N-expressed sample (Figure 2C). These results showed that SR4N expression was activated by p50-N interaction.

To gain more insights into the SR4N expression pattern, we analyzed its expression in our previously published RNA sequencing data from 3-week and 6-week-old *N. tabacum* leaves [27]. The FPKM values of NtaSR4Na (tobacco SR4Na) and NtaSR4Nb (tobacco SR4Nb) were about 106 and 79 in the 3-week-old leaf samples and 62 and 43 in the 6-week-old leaf samples, respectively (Figure 2D). Thus, the expression of both SR4N members decreased as the plants grew from young seedlings to maturity.

### 3.3. SR4N Is Required for N-p50 Triggered Cell Death

**Figure 3:**
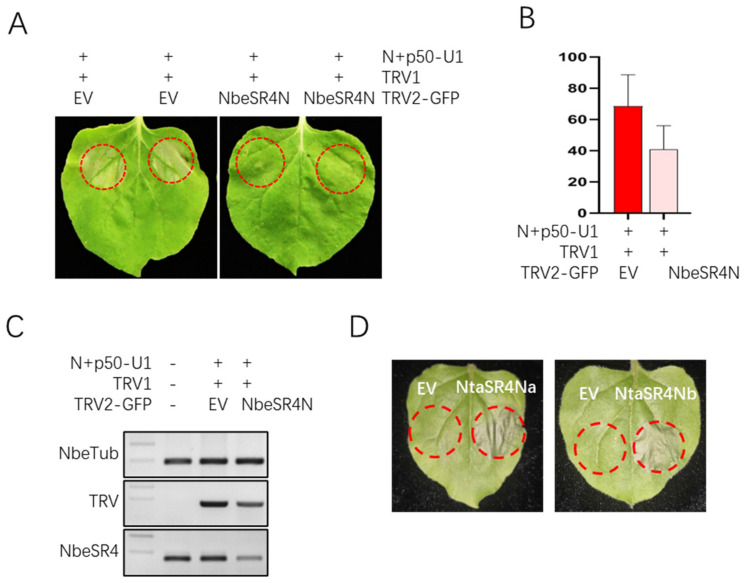
SR4N positively regulates cell death. (**A**) Cell death phenotype triggered by co-infiltration of N and p50 on *N. benthamiana* plants treated by control (left) and SR4N (right) VIGS vectors. Red circles mark the patches co-infiltrated with N and p50 Agrobacteria. (**B**) Statistical analysis of the cell death rate in the plants treated by control (left) and SR4N (right) VIGS vectors. Patches with HR/total infected patches indicate the cell death rate. A two-tailed Student’s t-test was used for comparison between the two samples using GraphPad Prism 8. (**C**) RT-PCR analysis of SR4N expression levels in VIGS experiments. Target genes are indicated to the left, and samples are indicated at the bottom of each lane. WT: wild-type untreated sample. (**D**) Cell death phenotype on SR4N overexpressed leaves. Infiltrated patches are marked by red circles, and vectors used for infiltration are indicated above each circle.

To test if SR4N participates in *N*-mediated resistance signaling, a virus-induced gene-silencing experiment was conducted. For this purpose, about 200 nt of SR4N cDNA was cloned into the TRV2-GFP vector, which expresses the GFP protein and enables the visual tracking of virus movements in plants. Three-week-old *N. benthamiana* plants were co-infiltrated with TRV1 and TRV2-GFP-SR4N or a TRV2-GFP control. Two weeks later, the upper leaves, which showed a GFP signal before, were co-infiltrated with agrobacterium containing *N* and *p50* constructs. Five days after the second infiltration, cell death appeared in about 70% and 40% of the infiltrated patches in TRV2-GFP and TRV2-GFP-SR4N, respectively (Figure 3A,B). An analysis of SR4N transcript levels using RT-PCR showed a clearly lower level of SR4N in TRV2-GFP-SR4N-treated plants compared with that of TRV2-GFP-treated or -untreated control plants (Figure 3C). These data showed that silencing SR4N impaired *N-p50*-triggered cell death, indicating SR4N is a positive regulator in cell death signaling. To further test this, two SR4N homologs were cloned into the overexpression vector and transiently expressed in *N. benthamiana* leaves. Interestingly, both SR4Na and b triggered strong cell death when overexpressed alone (Figure 3D). This result further confirmed that SR4N is a positive regulator of cell death in plants.

### 3.4. SR4N Negatively Regulates N-Independent and N-Mediated Immunity

**Figure 4:**
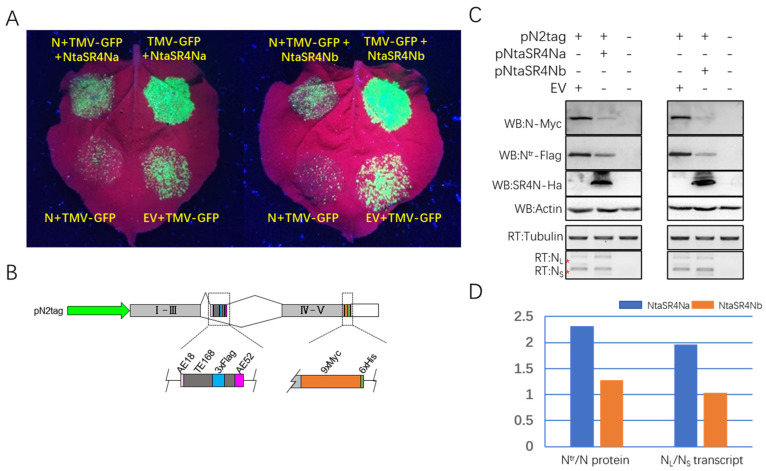
NtaSR4N promotes TMV replication but reduces N protein accumulation. (**A**) Green florescence imaging of agrobacteria-infiltrated *N. benthamiana* leaves under UV light. White circles mark the infiltrated patches with co-expressed genes indicated next to each circle. (**B**) Structure of *N* gene in pN2tag construct. Green arrow represents the native N promoter. Gray boxes marked I–III and IV–V represent fused exons I–III and exons IV–V of the wild-type *N* gene. The dashed box marks the modified alternative exon and 3′ ORF: AE18 and AE52 represent the 5′ 18- and 3′ 52-bp sequences of the 70-bp AE of wild = type *N*; TE168 represents a 168-bp SINE element from the tobacco genome (Supplementary Data S1); 3×FLAG, 9×Myc, and 6×His represent three-times Flag, nine-times Myc, and six = times His epitope tags, respectively. The open box represents the 3′ GRS of *N*. (**C**) Western blot and RT-PCR analysis of products from *N* and other genes in co-infiltration experiments. Constructs used in each sample are indicated on top of each lane. Target proteins are indicated to the left of the Western blots (top four panels). Target genes for RT-PCR analysis are indicated to the left of each gel image (bottom two panels). The two red * mark the position of N_L_ and N_S_ transcripts, respectively. (**D**) Quantification of N^tr^/N ratio at the protein level (left) and N_L_/N_S_ ratio at the transcript level (right). Blue columns represent data from NtaSR4Na-infiltrated samples, and brown columns represent data from NtaSR4Nb-infiltrated samples.

Next, we asked how SR4N affects plant immunity against TMV in the absence or in presence of *N*. To answer this question, a four-patch infiltration was conducted on *N. benthamiana* leaves. An N and TMV-GFP agrobacterium mixture, empty vector (EV), and TMV-GFP mixture; an N, TMV-GFP, and SR4N mixture; and a TMV-GFP and SR4N mixture were infiltrated on the same leaf (Figure 4A). As expected, the EV+TMV-GFP-infiltrated patch showed bright green florescence, and N+TMV-GFP clearly showed restricted green florescence due to *N*-mediated immunity against TMV. To our surprise, the addition of SR4N resulted in an increase in green florescence signal regardless of whether *N* was present or not, and both homologs had similar effects (Figure 4A). These results suggested that SR4N plays a negative role in *N*-independent plant immunity against TMV.

The increase in green florescence caused by SR4N in the presence of *N* could be due to the downregulation of either *N*-independent immunity or *N*-mediated immunity. Since SR4N showed interaction with the *N* terminator and promoter, we tested whether SR4N directly regulates *N* expression, alternative splicing, or both. To answer this question, a double-tagged *N* construct, pN2tag, was generated (Figure 4B). In this vector, the minimal *N* containing the native promoter, constitutive exon 1–5, intron III with AE, and the terminator was modified such that a 3×Flag and 9×Myc-6×His tags were in-frame fused to the C-termini of truncated N^tr^ and full-length N, respectively (Figure 4B). To test the impact of SR4N on *N* expression, a pN2tag agrobacterium was co-infiltrated with agrobacteria containing EV, or SR4N in *N. benthamiana* leaves. The infiltrated tissue was harvested 36 h post-infiltration, and protein and RNA were prepared from these samples. Western blot analyses using Myc and Flag antibodies detected clear, specific bands corresponding to the predicted size of N and the N^tr^ protein, and a specific band corresponding to SR4N protein was also detected using HA antibody (Figure 4C, 3rd panel). Interestingly, the intensity of both the N and N^tr^ bands was significantly reduced in samples with SR4N expression compared to those with EV (Figure 4C, 1st and 2nd panels), while the overall transcript level did not change significantly (Figure 4C, bottom panel). These results indicated that SR4N negatively regulates N-mediated immunity by inhibiting the translation of N transcripts or promoting the degradation of N proteins. A quantitative analysis of the signal intensity revealed that the SR4N expression clearly increased the ratio of N^tr^/N at the protein level, while the ratio of N^tr^/N at the transcript level was only slightly increased by the SR4N expression (Figure 4D), indicating SR4N has a differential effect on N^tr^ and N at the protein level and the transcript level.

### 3.5. SR4N Is Conserved in Plants

**Figure 5:**
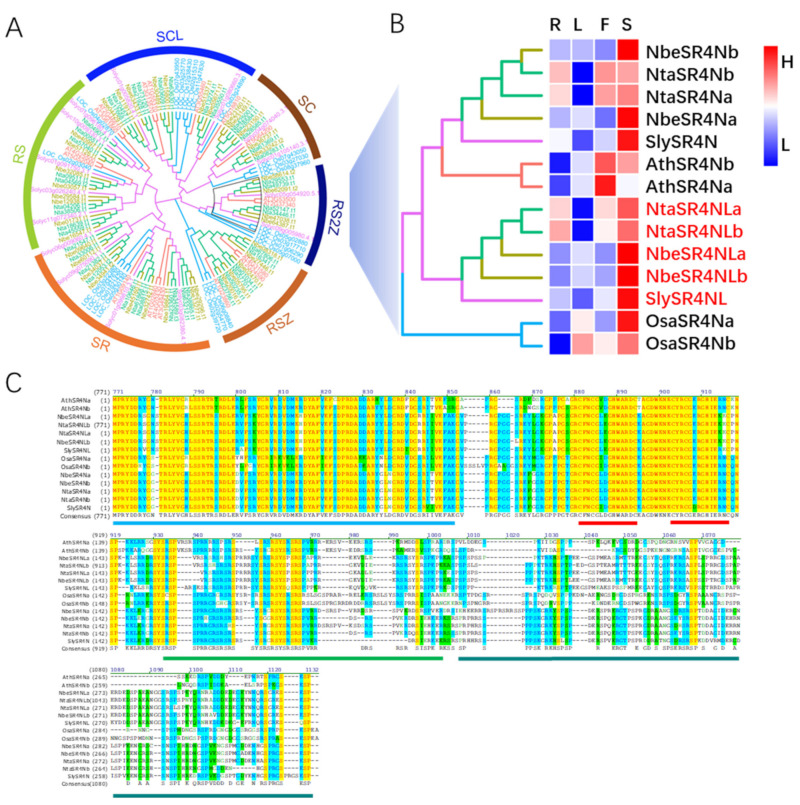
SR4N is conserved in both monocots and dicots. (**A**) Phylogeny tree of SR protein family from rice (blue IDs), Arabidopsis (orange IDs), tomato (purple IDs), and tobacco (green IDs) genomes. (**B**) Expression heatmap of RS2Z subclade members. Published RNA-seq data were obtained and analyzed (see Section 2.6). Sample sources are indicated on top of the heatmap: R, root; L, leaf; F, flower; S, seed. Gene IDs are indicated to the right. (**C**) Protein sequence alignments for the RS2Z subclade proteins. Blue line marks the RRM motif; red lines mark the zinc finger motifs; green line marks the serine/arginine-rich region; dark blue line marks the serine/proline-rich region.

To find out whether SR4N is conserved in plants, we retrieved all serine/arginine-rich proteins from the Arabidopsis, tobacco, tomato, and rice proteome and conducted a phylogeny analysis (see Section 2.8). The results showed that all of these SR proteins can be divided into six subfamilies, which is consistent with the previous classification [39]. SR4N belongs to the RS2Z subfamily, which contains four tobacco members, two tomato members, two Arabidopsis members, and four rice members (Figure 5A). In dicots, these RS2Z proteins formed two subclades, SR4N and SR4N-like (SR4NL). The former has two members in tobacco (NtaSR4Na and NtaSR4Nb), two members in *N. benthamiana* (NbeSR4Na and NbeSR4Nb), one member in tomato (SlySR4N), and two members in Arabidopsis (AthSR4Na and AthSR4Nb). The latter was lost in Arabidopsis and has two members in tobacco (NtaSR4NLa and NtaSR4NLb), two members in *N. benthamiana* (NbeSR4NLa and NbeSR4NLb), and one member in tomato (SlySR4NL). The two rice members that shared higher identities with SR4N were named OsaSR4Na and OsaSR4Nb (Figure 5A).

The expression patterns of all these SR4N and SR4NL homologs were analyzed using RNA-seq data (see Section 2.5). The expression levels of all these proteins in root, leaf, flower, and seed samples were determined by normalizing the actin mRNA expression level in the same database. The overall expression levels for different SR4N and SR4NL homologs were highest in seeds and lowest in leaves (Figure 5B), indicating they may play essential roles in seed development in both dicots and monocots. Considering cell death caused by SR4N overexpression, the low expression level of SR4N in leaves may reflect a balancing mechanism between resistance and development.

It was reported that the RS2Z subfamily protein has an N-terminal RNA recognition motif (RRM), followed by two CxxCxxxxHxxxxC zinc finger motifs, a serine/arginine-rich region, and a serine/proline-rich region at the C-terminal [39]. A protein sequence alignment showed that all the tobacco and tomato RS2Z subfamily proteins consist of these five motifs; however, one tobacco member, NtaSR4NLa, contains additional motifs in the N-terminals (Figure 5C). The N-terminal sequences of NtaSR4NLa were analyzed using the online webserver motifscan [40] and ABC_transporter 1 and ABC-2-type transporter motifs were identified (Figure S2). The presence of all these conserved motifs in SR4N and SR4NL homologs indicated that these proteins had conserved functions and mechanisms of action.

### 3.6. Roles of SR4N on N-Independent and N-Mediated Immunity Are Conserved

**Figure 6:**
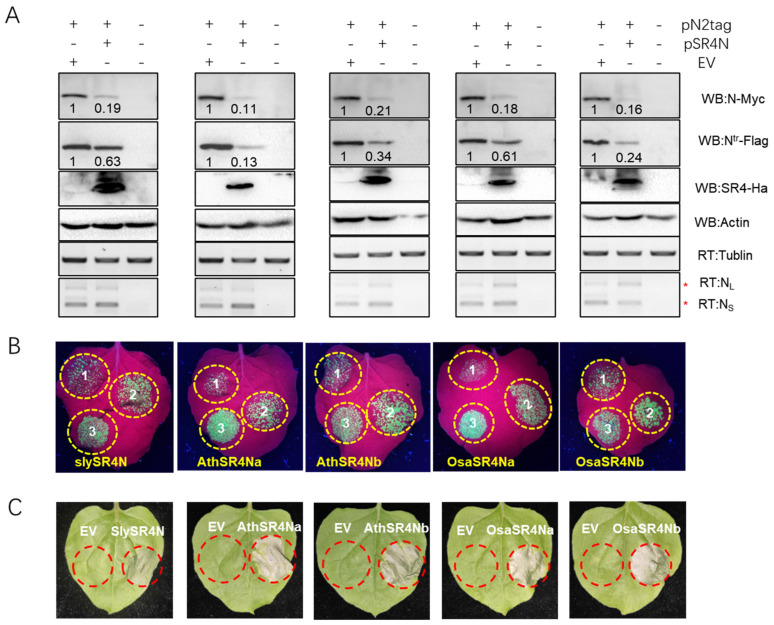
SR4N functions are conserved in homologs from different plant species. (**A**) Impact of tomato, Arabidopsis, and rice SR4N on N protein and transcript accumulation. Co-expressed constructs are indicated on top of each lane. The SR4Ns expressed from the left to the right are SlySR4N, AthSR4Na, AthSR4Nb, OsaSR4Na, and OsaSR4Nb. Top four rows are Western blots, with target proteins indicated to the right. Bottom two rows are RT-PCR gels, with target transcripts indicated to the right. Red stars * mark the N_L_ and N_S_ isoforms. (**B**) Green florescence imaging of leaves in co-infiltration experiments. Yellow circles mark the infiltrated patches. Circle 1 is co-infiltrated with TMV-GFP and N; circle 2, TMV-GFP and EV; circle 3, TMV-GFP, N, and SR4N, indicated at the bottom. (**C**) Cell death triggered by SR4Ns from different plant species. Red circles mark the infiltrated patches, with vectors indicated above.

In order to investigate whether SR4N genes from other plant species have similar functions to *NtaSR4N* in plant immunity, *SlySR4N*, *AthSR4Na* and *b*, and *OsaSR4Na* and *b* were cloned from tomato, Arabidopsis, and rice plants into expression vectors (see Section 2.2). First, their roles in *N* gene expression and N protein accumulation were tested in co-infiltration experiments. A Western blot analysis showed that all these SR4N homologs significantly reduce N and N^tr^ protein accumulation and have stronger effects on N than N^tr^ (Figure 6A). RT-PCR experiments were conducted to detect the N_L_ and N_S_ transcript levels, and the results showed that overall transcript levels were not significantly changed by the co-expression of these SR4Ns. The rice SR4Na and b expressions slightly increased the ratio of N_L_/N_S_, but the other ones did not (Figure 6A). These results showed that SR4Ns from different species have a conserved role in negatively regulating N protein levels, and the rice homologs may have a minor role in regulating the AS of N-like NtaSR4Na.

Next, the roles of various SR4Ns on N-mediated TMV resistance were tested with co-infiltration experiments. TMV-GFP was co-expressed with *N* (Figure 6B, circle 1), EV (Figure 6B, circle 2), and *N*, plus various SR4Ns (Figure 6B, circle 3). Green florescence imaging under UV light showed that the GFP signal was clearly weaker in patch 1 than in patch 2, while it was stronger in patch 3 than in patch 1 (Figure 6B), indicating N effectively restricted TMV-GFP accumulation when expressed alone, while N-mediated immunity against TMV-GFP was compromised by all tested SR4Ns, particularly *AthSR4Na* and *OsaSR4Na*.

The overexpression of SR4Ns was also analyzed in transient assays in *N. benthamiana* leaves. The results showed that the overexpression of all tested SR4N homologs resulted in clear cell death in the infiltrated patches (Figure 6C), indicating all these SR4Ns are positive regulators of cell death like the *NtaSR4Na* and *b*.

In summary, the above experiments showed that SR4N gene functions on N expression, N-mediated immunity, and cell death regulation are conserved among both dicots and monocots, suggesting that SR4N is a highly conserved regulator of plant immunity.

### 3.7. SR4Ns Promote Multiple Virus Replication but Not Transgene Expression

**Figure 7:**
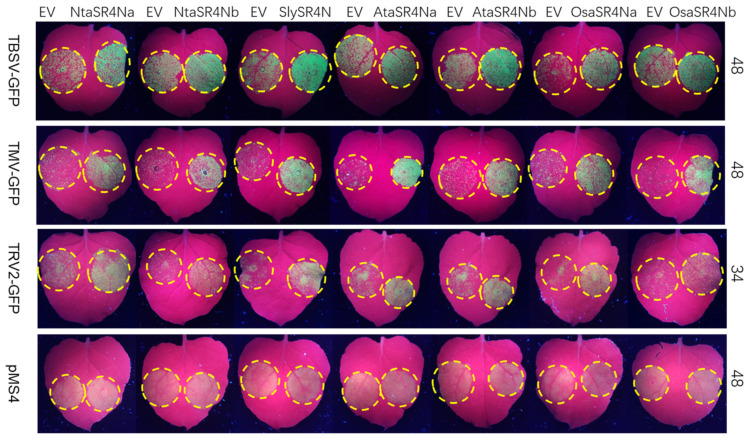
SR4N promotes the replication of multiple viruses but not the expression of the transgene. Yellow dashed circles mark the infiltrated patches. The viruses indicated to the left of each row and EV or SR4N indicated on top of each leaf were co-infiltrated. Images were taken at timepoint HPI are indicated to the right of each row. TBSV-GFP agrobacterium was infiltrated at a final OD600 of 0.02, TMV-GFP at 0.5, TRV1 and TRV2-GFP at 0.01, pMS4 at 0.05, and EV and SR4N agrobacteria at 0.1.

The promotion of TMV-GFP replication by NtaSR4Na and b raises the questions of whether they have a general role in other virus replications, whether this role is conserved in other plant species, and whether it is related to the suppression of siRNA-mediated silencing. To answer these questions, all SR4N homologs were tested with TBSV-GFP, TMV-GFP, and TRV2-GFP in a half-leaf assay. An agrobacterium containing each virus was co-infiltrated with EV control on one side of the leaf and with different SR4Ns on the other side (Figure 7). GFP imaging at 34 to 48 HPI showed that the co-expression of all SR4Ns enhanced the GFP florescence signal compared with the EV control (Figure 7, top three rows), indicating SR4N has a conserved, general role in promoting viral replication. As a suppressor of RNA silencing can promote heterologous virus replications [41] and enhance transgene expressions in transient assays, we tested whether these SR4Ns can enhance the expression of 35S-GFP. For this, a pMS4 vector-encoding 35S-GFP transcription unit was co-infiltrated with EV and SR4N vectors side by side. The results showed that none of the tested SR4Ns had a visible effect on GFP florescence compared with EV (Figure 7, bottom row), suggesting SR4N may not have a role in silencing suppression.

## 4. Discussion

NLRs play important roles in plant immunity yet they also impose potential threats to plant development; thus, the proper regulation of NLR expressions and functions is critical for the success of plants in evolution and for breeding crops to achieve a balance between disease resistance and optimal yields. In this work, using the N-TMV interaction as a model system, we studied the role of a class of RS2Z subtypes in SR proteins, which we named SR4N, in N regulation and plant immunity in general. SR proteins have been identified as being involved in RNA processing, such as transcription; splicing; polyadenylation; mRNP packing in the nucleus for mRNA export, translation, and decay in the cytoplasm; and non-coding RNA processing [42,43,44,45]. NtaSR4Na was identified through its interaction with *N* gene 3′GRS fragments (Figure 1). The co-expression of NtaSR4Na and OsaSR4Na slightly increased the ratio of N_L_/N_S_ and N^tr^/N (Figure 4 and Figure 6A). These data indicated that SR4N plays a possible role in regulating the AS of *N*. This is consistent with the role of splicing regulation. In the classic model, SR protein regulates splicing by binding to splicing enhancer or repressor elements in pre-mRNA, so it seemed contradictory for SR4N to play a role in the AS of N by binding to its 3′GRS DNA. Interestingly, it was shown in a previous study that the production of N_L_ transcript is dependent on 3′GRS of *N* [23]. The Arabidopsis AthSR4Na gene was shown to enhance the usage of alternative splicing sites when overexpressed [46]. Thus, it is plausible that SR4N may recruit splicing machinery to *N* by interacting with 3′GRS to enhance AS. This is consistent with the “chromatin–adaptor” model of alternative splicing [47]. The role and mechanism of NtaSR4Na on the AS of *N* would be worthwhile for further investigation.

Besides potential roles in regulating the AS of *N*, SR4N from all tested plant species clearly reduced overall N protein levels and promoted TMV-GFP replication when co-expressed with *N* and TMV-GFP (Figure 4A,C and Figure 6A,B), suggesting SR4N has a conserved negative regulatory role in N-mediated immunity. Since the overall N_L_/N_S_ transcript levels did not significantly change when SR4N was co-expressed (Figure 4C and Figure 6C), SR4N may reduce the translation efficiency of *N* transcripts or promote N protein stability. Several mechanisms are possible for SR4N’s effect on *N* expression. SR4N may affect *N* mRNA translation by interfering in its mRNA polyadenylation and export considering it binds to its 3′GRS. It may reduce N translation or protein stability indirectly by regulating other factors in translation or protein degradation. It would be important to determine whether this effect on N is dependent on its interaction with its 3′GRS and specific for *N* or general for NLR.

In this study, we also found that NbeSR4N was required for N-p50-triggered cell death via the VIGS knockdown of NbeSR4N, and further experiments showed that the overexpression of SR4N alone can trigger cell death (Figure 3 and Figure 6C). These results suggested that SR4N is a positive regulator of ETI-triggered cell death pathways and functions downstream of immune receptor activation. Our study also revealed that SR4N’s expression enhanced multiple virus replications in wild-type *N. benthamiana* plants (Figure 4A and Figure 7) but did not increase transgene expression from 35S-GFP, which indicated that SR4N may not be an endogenous silencing suppressor. A role in the suppression of siRNA-mediated silencing might also be contradictory with SR4N’s negative role in N protein accumulation, as *N* is repressed by miRNAs [16]. These contradictions indicated that SR4N may be involved with a novel mechanism in plant-virus interactions.

The functions of the SR4N family genes are understudied in plants. The overexpression of Arabidopsis AthSR4Na causes pleiotropic developmental phenotypes, affecting cell shape, stomata development, the formation of meristems, auxin signaling, in vitro regeneration, etc. [46]. The expression of AthSR4Na has also been shown to be responsive to drought stress [48]. Thus, our study, in conjunction with previous studies, revealed that SR4N genes play important roles in plant development and abiotic and biotic stress responses, and these await further investigations.

## Data Availability

Not applicable.

## Supplementary Materials

The following supporting information can be downloaded at: https://www.mdpi.com/article/10.3390/v15010026/s1, Figure S1: Yeast one hybrid screen, Figure S2: Motif scan results for NtaSR4NLa, Table S1: Oligonucleotides used in this study, Data S1: Sequences of vectors used in this study, Data S2: SR family protein sequences from tomato, tobacco, Arabidopsis, rice and *N. benthamiana*.
