# Supplementary material for: A Conserved, Serine-Rich Protein Plays Opposite Roles in N-Mediated Immunity against TMV and N-Triggered Cell Death
Source: Viruses. 2022 Dec 21;15(1):26. doi: 10.3390/v15010026 (PMC9865399; doi:10.3390/v15010026)
Supplement: Supplementary file 1 [file viruses-15-00026-s001.zip › viruses-2023737-supplementary.pdf]

A

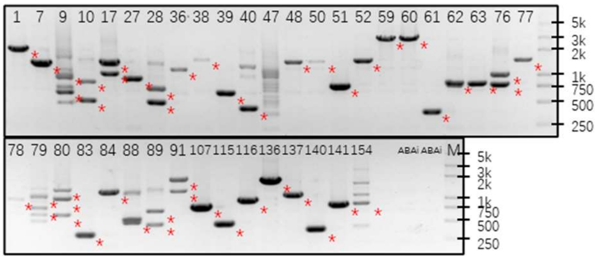

B

No.	Tobacco coding gene	Nr annotation
28-750	Nta65245	60S ribosomal protein L10 (LOC107827878)
36-1200	Nta25888/Nta14609	60S ribosomal protein L18a-like protein (LOC107825033)
1-2000	Nta63440/Nta03459	Coatomer subunit gamma (LOC104241872)/包被体
38/50/77-1500	Nta52339	Ceramide Inositol phosphorylceramide synthase 2(LOC107774953)
62-750	Nta39263	Chlorophyll A/B binding protein 1
63-750	Nta39264/Nta39261	Chlorophyll A/B binding protein 1
76-1000	Nta36680	Chlorophyll A/B binding protein 1
91-1300	Nta25862	Chlorophyll A/B binding protein 1
89-300	Nta38778	CLP protease regulatory subunit CLPX1
89-750	Nta28791	Fructose-1,6-bisphosphatase (LOC107807379)
154-750	Nta46861	GDT1-like protein 2 , chloroplastic (LOC107769454)
84-1500	Nta66165	Oxygen-evolving enhancer protein 1, chloroplastic-like (LOC107786842)
27/116-1000	Nta12980/Nta35723	1,2-dihydroxy-3-keto-5-methylthiopentene dioxygenase 2 (LOC107828443)
137-1500	Nta49739	serine/arginine-rich splicing factor RS2Z33-like (LOC107812417)
7-1500	Nta03851/Nta38089	UDP-glucose-1-phosphate uridylyltransferase 3 (LOC104085415)
52/80-1300	Nta58346	zinc finger CCCH domain-containing protein 20-like (LOC107771097)
80-1000	Nta42125/Nta50003	Zinc finger CCCH domain-containing protein ZFN-like (LOC107764729)

C

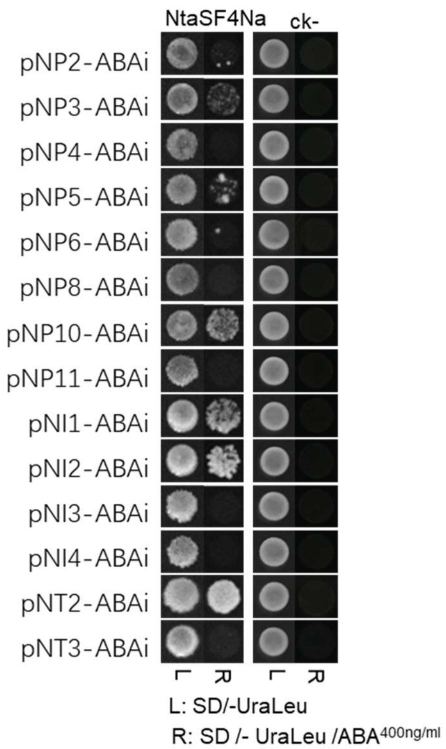

Figure S1. Yeast one hybrid screen for *N*-interacting proteins from tobacco cDNA library. (**A**) PCR amplification of cDNA insertions from selected yeast clones. DNA bands marked by red star to their right were gel purified and sequenced. DNA ladders were marked on the right. Red stars marked the DNA bands purified for sequencing analysis. (**B**) Annotation of amplified insertion sequences. (**C**) Point-to-point Y1H test between NtaSF4Na and each bait vector. Medium composition L (SD/-Ura -Leu) and R (SD/-Ura -Leu/ABA$^{400\ ng/mL}$) were indicated at the bottom. Bait vector IDs were indicated to the left and prey vectors are indicated on top.

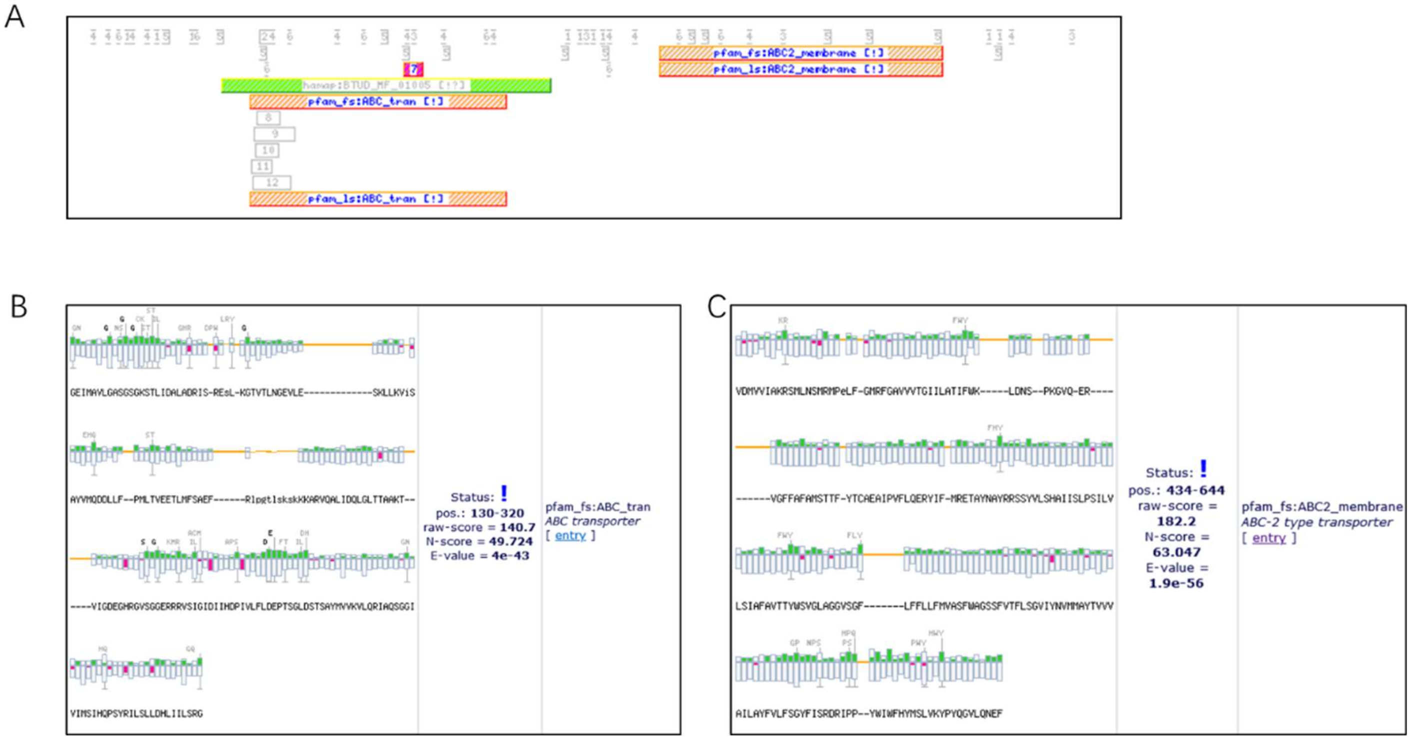

Figure S2. Motif scan results of NtaSF4NLa N-terminal region. (**A**) The distribution of the detected motifs in the N-terminal 770 amino acids of NtaSF4NLa. (**B**) The details of the ABC transporter domain. (**C**) The details of the ABC-2 type transporter domain.

Table S1. Oligonucleotides used in this work.

Primer ID	Sequence	Experiment	Target	Description	vector name
ZQLP1136	TTCGAGCTCTCGAG-GATCTTATTCTAATTATATGAC		N promoter fragment1(NP1)		
ZQLP1137	TGCCTCGAGGGAGTTCCAG-CATAATGTACTG				
ZQLP1134	TTCGAGCTCGTCAACGTTT-GTCAAGTCATTG		N promoter fragment2(NP2)		
ZQLP1135	TGCCTCGAGCCAGAT-TTACAAGTGCTCATTCA				
ZQLP1132	TTCGAGCTCGGATCTT-GAACAATGTGTTCG		N promoter fragment3(NP3)		
ZQLP1133	TGCCTCGAGGCCTAGAATGCTGAAC-CTTTG				
ZQLP1130	TTCGAGCTCGGCTTTTGCATCTA-TATCCACT		N promoter fragment4(NP4)		
ZQLP1131	TGCCTCGAGGCCTTATTT-GATCGCTCTCA				
ZQLP1128	TTCGAGCTCCAGGTATTAGAAAGGTGGCG		N promoter fragment5(NP5)		
ZQLP1129	TGCCTCGAGATTGGTCGCTA-TATTGGTGGT				
ZQLP1126	TTCGAGCTCTATACCGACCGAAGTT-GGTC		N promoter fragment6(NP6)		
ZQLP1127	TGCCTCGAGTT-GGAGCCAGGGATTAGTG				
ZQLP1124	TTCGAGCTCTACACACGTAACAC-GCTTGC		N promoter fragment7(NP7)	various N promoter fragment for Y1H	
ZQLP1125	TGCCTCGAGGCGCAGATA-GAAAGTGACAAC	PCR from SPDK450 vector			pABAi
ZQLP1122	TTCGAGCTCAATTCGGCAAGAA-TAATCAATT		N promoter fragment8(NP8)		
ZQLP1123	TGCCTCGAGTCGCCTCTTCGTAA-TAGTGC				
ZQLP1120	TTCGAGCTCCTGGAGTAAACATTAA-GATGAAGC		N promoter fragment9(NP9)		
ZQLP1121	TGCCTCGAGGGCATCACTTGGTGCG-TATT				
ZQLP1118	TTCGAGCTCGCATACACTG-CATTATGCACTAG		N promoter fragment10(NP10)		
ZQLP1119	TGCCTCGAGGGTCTCCATT-GATTGAATTCTT				
ZQLP1116	TTCGAGCTCACATGTGATCATTCAACTTTGTG		N promoter fragment11(NP11)		
ZQLP1117	TGCCTCGAGGGACTCAAC-GTTAATTCTCTGAA				
ZQLP1114	TTCGAGCTCCAGAAACAAAGGTACAATAGCTTG		N intronIII fragment1 (NI1)		
ZQLP1115	TGCCTCGAGCATTTGCATTATTT-GACAAATTT				
ZQLP1112	TTCGAGCTCTTGGCTA-GAATTATGATACATGTCT		N intronIII fragment2 (NI2)	various N intron fragment for Y1H	
ZQLP1113	TGCCTCGAGTTCTTGTAAGAAGAA-TATTGACCG				

ZQLP1110	TTCGAGCTCTTTCATTCGTT-GTACTGAATGC		N intronIII fragment3 (NI3)		
ZQLP1111	TGCCTCGAGGTGAGTTA-GAAAACTCCCGTAGAG				
ZQLP1108	TTCGAGCTCTTCAGTCCAATAA-GAATTCAATTT		N intronIII fragment4 (NI4)		
ZQLP1109	TGCCTCGAGTAC-GAGGGCCAACACATTAT				
ZQLP1106	TTCGAGCTCGACGAACGACATTAA-GAGCC		N intronIII fragment5 (NI5)		
ZQLP1107	TGCCTCGAGCTGTTTAGAACACAGA-CAGAATGAG				
ZQLP1104	TTCGAGCTCTGATGTACAT-ATCAACAACGAGTT		N terminator framgent1(NT1)		
ZQLP1105	TGCCTCGAGGGTG-CAATCGGGCTAACTAC				
ZQLP1102	TTCGAGCTCGCCTTT-GCTGACAAGTAACC		N terminator framgent2(NT2)	various N terminator fragment for Y1H	
ZQLP1103	TGCCTCGA-GATGGATCTCTTAATGGTGCG				
ZQLP1100	TTCGAGCTCTTACTGTTGGGCTAAAC-TACCC		N terminator framgent3(NT3)		
ZQLP1101	TGCCTCGAGAAGCTTCTG-CAGGTCGAGT				
ZQLP1749	gcttacgccgaggtcATGCCGCGG-TATGATGATAG		Nta49739	tobacco SR4Na gene for Y1H assay	pGADT7LIC2.0
ZQLP1750	atttagggaagag-gAGGTGACTCGCTGCCCCT				
ZQLP1775	gattacgccgaggtcATGCCGCGG-TATGATGATAG		Nta49739	tobacco SR4Na gene for expression	
ZQLP1777	atcatagggaagag-gAGGTGACTCGCTGCCCCTTGGA-GAAGC				
ZQLP1775	gattacgccgaggtcATGCCGCGG-TATGATGATAG		Nta29553	tobacco SR4Nb gene for expression	
ZQLP1776	atcatagggaagag-gAGGTGACTCGCTGCCCCTTGGA-GAACC				
ZQLP1695	gattacgccgaggtcATGCCGCGG-TATGATGACA		Solyc05g054920.5	tomato SR4N gene for expression	
ZQLP1696	atcatagggaagaggGGGTGACTCAC-TGCCCCTT	RT-PCR			
ZQLP1727	gattacgccgaggtcATGCCTCGC-TATGATGATCG		AT2G37340	Arabidopsis SR4Na gene for expression	pH7LIC8.1
ZQLP1728	atcatagggaagaggAGGAGACTCAC-TTCCTCTAGGG				
ZQLP1727	gattacgccgaggtcATGGCAGCGATTAC-GCAGA		AT3G53500	Arabidopsis SR4Nb gene for expression	
ZQLP1729	atcatagggaagaggAGGTGACTCAC-TGCCTTTAGG				
ZQLP1769	gattacgccgaggtcATGCCGCGC-TATGATGATCGT		LOC_Os05g02880	Rice SR4Na gene for expression	
ZQLP1770	atcatagggaagaggGGCTTCAGGA-GACTCGCTCG				
ZQLP1771	gattacgccgaggtcATGCCTCGC-TATGATGATCAT		LOC_Os03g17710	Rice SR4Nb gene for expression	
ZQLP1772	atcatagggaagaggAGCTTCAGGG-GACTCGCTTG				

ZQLP1643	gaggagaagagcccGTAC-CTCGTGGTCCAGGTGG		Nbe58614/Nbe62091	*N. benthamiana* VIGS experiment	TRV2-GFP
ZQLP1644	cgacgacaagacccTCCAATTTCTTGGGGC-TATT				
ZH1010	ACTATCTATTCGATGATGAAGA-TACCC	colony PCR	tobacco cDNA library	Yeast one hybrid screen	pGADT7-rec
ZH1011	ACGATGTATAAATGAAAGAAATT-GAGA				

Data S1. Sequences of Vectors used in this study.

>pH7LIC8.1-Nta49739 1..867 35S promoter;881..1828 Nta49739(NtaSF4Na) coding sequences;1841..1930 3xHA tag sequence;1944..2169 35S terminator

AGATTAGCCTTTTCAATTTCAGAAAGAATGCTAACCCACAGATGGTTAGAGAGGCTTAC-
GCAGCAGGTCTCATCAAGACGATCTACCCGAGCAATAATCTCCAGGAAATCAAATACCTTCCCAAGAAGGTTAAAGATGC
AGTCAAAAGATTCAGGACTAACTGCATCAAGAACACAGAGAAAGATATATTTCTCAA-
GATCAGAAGTACTATTCCAGTATGGACGATTCAAGGCTTGCTTCACAAACCAAGGCAAGTAATAGAGATTGGAGTCTCTA
AAAAGGTAGTTCCCACTGAATCAAAGGCCATGGAGTCAAAGATTCAAATAGAGGAC-
CTAACAGAACTCGCCGTAAAGACTGGCGAACAGTTCATACAGAGTCTCTTACGACTCAATGACAAGAAGAAAATCTTCGT
CAACATGGTGGAGCACGACACACTTGTCTACTCCAAAAATATCAAAGATACAGTCTCAGAA-
GACCAAAGGGCAATTGAGACTTTTCAACAAAGGGTAATATCCGGAAACCTCCTCGGATTCCATTGCCCAGCTATCTGTCAC
TTTATTGTGAAGATAGTGGAAAAGGAAGGTGGCTCCTACAAATGCCATCATTGCGA-
TAAAGGAAAGGCCATCGTTGAAGATGCCTCTGCCGACAGTGGTCCCAAAGATGGACCCCCACCCACGAGGAGCATCGTG
GAAAAAGAAGACGTTCCAACCACGTCTTCAAAGCAAGTGGATTGATGTGATATCTCCAC-
TGACGTAAGGGATGACGCACAATCCCACTATCCTTCGCAAGACCCTTCCTCTATATAAGGAAGTTCATTTCATTTGGAGAG
AACACGGGGGACTCTAGAGGATCCAAGGAGATATAACATTACGCCGAGGTCATGCCGCGG-
TATGATGATAGGTATGGTGGTACACGCCTCTATGTTGGACATTTGTCTTCCAGAACGCGATCTCGAGACTTGGAGGACGTC
TTCAGCAGATACGGGAGAGTACGTGATGTGGATATGAAGCGTGACTATGCTTTTGTG-
GAATTTAGTGATCCTCGAGATGCTGATGATGCAAGATATGGCCTAAATGGGCGAGACGTTGATGGAAGCCGTATTACTGT
GGAATTCGCAAAAGGGGTACCTCGTGGTCCAGGTGGATCTCGAGAGTTTGGCGG-
CAGAGGTCCTCCTCCAGGTACAGGTCGTTGCTTTAATTGTGGACTTGATGGCCATTGGGCTCGAGATTGTAAGGCTGGGGA
CTGGAAGAACAAGTGTTACCGCTGTGGGGAGCGAGGTCATATAGAAAGAAACTGTCAGAA-
TAGCCCCAAGAAATTGAAACGTGGACGAAGTTATTCCCGCTCACCGTCTCCTCGTCGTGGCAGAAGTCGCAGCCACAGTT
ACAGCAGAGGTCGTAGCTACAGCCGATCCAGGTCTCCTGTGAG-
GAGGGACTCCAGGTCTCCTGTGAAGAGCGACTCCAGGTCTCCTGTGAAGAGGGACCGTAGCATTGAGCATGAAGAAAGA
AGATCAAGAAGTCCTCGCCCTCATAGATCATCACCAC-
CTCCATCAAAAGGAAGGAAATACAGCCCTTCACCTGATGAAAGAAGTCCACAAGAGAGAGGTACGCCGTCCCCTAGGGG
TGATAGGGCAGCCAATGGTTCTGATCATAGCAGGAGCCCTACAGACGATGCTGGAATAGAC-
GAACGCAGAAATTTGAGTCCTATTGAAGAAAATGGCCGCAGTCGCAGCAATAGCCCCATCCATAGGGACAATGGAAGCC
CAGTGGAGAATGGGAGCCCAATGGGTGATGATGAAAATCATGCTTCTCCAAGGGGCAGCGAG-
TCACCTCCTCTTCCCTATATGTCGCGTTATCCTTATGATGTGCCAGATTATGCATATCCATATGATGTTCCTGATTATGCATA
TCCATATGATGTTCCAGATTATGCTTAATGATATCCCGCGGCCATGCTAGAGTCCG-
CAAAAATCACCAGTCTCTCTCTACAAATCTATCTCTCTCTATTTTTCTCCAGAATAATGTGTGAGTAGTTCCCAGATAAGGG
AATTAGGGTTCTTATAGGGTTTCGCTCATGTGTTGAGCATATAAGAAACCCTTAG-
TATGTATTTGTATTTGTAAAATACTTCTATCAATAAAATTTCTAATTCCTAAAACCAAAATCCAGTGACCT

>pH7LIC8.1-Nta29553 1..867 35S promoter;881..1786 Nta29553 (NtaSF4Nb) coding sequences; 1799..1888 3xHA tag sequence; 1902..2127 35S terminator

AGATTAGCCTTTTCAATTTCAGAAAGAATGCTAACCCACAGATGGTTAGAGAGGCTTAC-
GCAGCAGGTCTCATCAAGACGATCTACCCGAGCAATAATCTCCAGGAAATCAAATACCTTCCCAAGAAGGTTAAAGATGC
AGTCAAAAGATTCAGGACTAACTGCATCAAGAACACAGAGAAAGATATATTTCTCAA-
GATCAGAAGTACTATTCCAGTATGGACGATTCAAGGCTTGCTTCACAAACCAAGGCAAGTAATAGAGATTGGAGTCTCTA
AAAAGGTAGTTCCCACTGAATCAAAGGCCATGGAGTCAAAGATTCAAATAGAGGAC-
CTAACAGAACTCGCCGTAAAGACTGGCGAACAGTTCATACAGAGTCTCTTACGACTCAATGACAAGAAGAAAATCTTCGT
CAACATGGTGGAGCACGACACACTTGTCTACTCCAAAAATATCAAAGATACAGTCTCAGAA-
GACCAAAGGGCAATTGAGACTTTTCAACAAAGGGTAATATCCGGAAACCTCCTCGGATTCCATTGCCCAGCTATCTGTCAC

TTTATTGTGAAGATAGTGGAAAAGGAAGGTGGCTCCTACAAATGCCATCATTGCGA-
TAAAGGAAAGGCCATCGTTGAAGATGCCTCTGCCGACAGTGGTCCCAAAGATGGACCCCCACCCACGAGGAGCATCGTG
GAAAAAGAAGACGTTCCAACCACGTCTTCAAAGCAAGTGGATTGATGTGATATCTCCAC-
TGACGTAAGGGATGACGCACAATCCCACTATCCTTCGCAAGACCCTTCCTCTATATAAGGAAGTTCATTTCATTTGGAGAG
AACACGGGGGACTCTAGAGGATCCAAGGAGATATAACATTACGCCGAGGTCATGCCGCGG-
TATGATGATAGGTATGGTGGTACACGCCTCTATGTTGGACATTTGTCTTCCAGAACGCGATCTCGAGACTTGGAGGACGTC
TTTAGCAGATACGGGAGAGTACGTGATGTGGATATGAAGCGTGACTATGCTTTTGTG-
GAATTTAGTGATCCTCGAGATGCTGATGATGCAAGATACGGCCTAAATGGGCGGGACGTTGATGGAAGCCGTATTACTGT
GGAATTCGCAAAAGGGGTACCTCGTGGTCCAGGTGGATCTCGAGAGTTTGGCGG-
CAGAGGTCCTCCTCCAGGTACAGGTCGTTGCTTTAATTGTGGACTTGATGGCCATTGGGCTCGAGATTGTAAGGCTGGGGA
CTGGAAGAACAAGTGTTACCGCTGTGGGGAGCGAGGTCATATAGAAAGAAACTGTCAGAA-
TAGCCCCAAGAAATTGAAACGTGGACGAAGTTATTCCCGCTCACCGTCTCCTCGTCGTGGCAGAAGTCGCAGCCGCAGTT
ACAGCAGAGGTCGTAGCTACAGCCGATCCAGGTCTCCTGTGAG-
GAGGGACTCCAGGTCTCCTGTGAAGAGGGACCAAAGCATTGAGCATGAAGAGAGAAGATCAAGTAGTCCTCGCCCTCGA
AGATCATCACCACCTCCATCAAAAGGAAGGAAATACAGCCCTTCACCTGATGAAA-
GAAGTCCACAAGAGAGAGGTACGCCGTCCCCTAAGGGTGATAGGGCAGCCAATGGTTCCGAGTATAGCAGGAGCCCTAC
AGACGATGCTGGAATAGACGAACGCAGAAATTTGAGCCCTATTGAAGAAAATGGCCGCAG-
TCACAGCAATAGCCCCATCCATAGGGAGAACGGGAGCCCAATGGGTGATGATGAAAATCATGGTTCTCCAAGGGGCAGC
GAGTCACCTCCTCTTCCCTATATGTCGCGTTATCCTTATGATGTGCCAGATTATGCATA-
TCCATATGATGTTCCTGATTATGCATATCCATATGATGTTCCAGATTATGCTTAATGATATCCCGCGGCCATGCTAGAGTCC
GCAAAAATCACCAGTCTCTCTCTACAAATCTATCTCTCTCTATTTTTCTCCAGAA-
TAATGTGTGAGTAGTTCCCAGATAAGGGAATTAGGGTTCTTATAGGGTTTCGCTCATGTGTTGAGCATATAAGAAACCCTT
AGTATGTATTTGTATTTGTAAAATACTTCTATCAATAAAATTTCTAATTCCTAAAACCAAAATCCAGTGACCT
>pH7LIC8.1-Solyc05g054920 1..867 35S promoter;881..1798 Solyc05g054920(SlySF4N) coding sequences; 1811..1900 3xHA tag sequence; 1914..2139 35S terminator
AGATTAGCCTTTTCAATTTCAGAAAGAATGCTAACCCACAGATGGTTAGAGAGGCTTAC-
GCAGCAGGTCTCATCAAGACGATCTACCCGAGCAATAATCTCCAGGAAATCAAATACCTTCCCAAGAAGGTTAAAGATGC
AGTCAAAAGATTCAGGACTAACTGCATCAAGAACACAGAGAAAGATATATTTCTCAA-
GATCAGAAGTACTATTCCAGTATGGACGATTCAAGGCTTGCTTCACAAACCAAGGCAAGTAATAGAGATTGGAGTCTCTA
AAAAGGTAGTTCCCACTGAATCAAAGGCCATGGAGTCAAAGATTCAAATAGAGGAC-
CTAACAGAACTCGCCGTAAAGACTGGCGAACAGTTCATACAGAGTCTCTTACGACTCAATGACAAGAAGAAAATCTTCGT
CAACATGGTGGAGCACGACACACTTGTCTACTCCAAAAATATCAAAGATACAGTCTCAGAA-
GACCAAAGGGCAATTGAGACTTTTCAACAAAGGGTAATATCCGGAAACCTCCTCGGATTCCATTGCCCAGCTATCTGTCAC
TTTATTGTGAAGATAGTGGAAAAGGAAGGTGGCTCCTACAAATGCCATCATTGCGA-
TAAAGGAAAGGCCATCGTTGAAGATGCCTCTGCCGACAGTGGTCCCAAAGATGGACCCCCACCCACGAGGAGCATCGTG
GAAAAAGAAGACGTTCCAACCACGTCTTCAAAGCAAGTGGATTGATGTGATATCTCCAC-
TGACGTAAGGGATGACGCACAATCCCACTATCCTTCGCAAGACCCTTCCTCTATATAAGGAAGTTCATTTCATTTGGAGAG
AACACGGGGGACTCTAGAGGATCCAAGGAGATATAACATTACGCCGAGGTCATGCCGCGG-
TATGATGACAGGTATGGTGGCACACGCCTATATGTTGGACATTTGTCTTCCCGGACGCGATCTCGAGACTTGGAGGACGTC
TTTAGCAGATATGGGAGAGTACGTGATGTGGATATGAAGCGTGACTATGCTTTTGTGGAG-
TTTAGTGATCCTCGAGATGCCGATGATGCAAGATACGGCCTAAATGGGCGAGATGTTGATGGAAGTCGTGTTATCGTGGA
GTTCGCCAAAGGGGTGCCTCGTGGTCCAGGTGGATCTCGAGAGTTTGGTGG-
CAGAGGTCCTCCTCCAGGTACTGGTCGTTGCTTTAATTGTGGAATTGATGGACATTGGGCTCGAGATTGTAAAGCTGGGGA
CTGGAAGAACAAGTGTTACCGCTGTGGAGACCGAGGTCATATAGAAAGGAACTGTCAGAA-
TAGCCCCAAGAAATTGAAACGTGACCGAAGTTATTCCCGCTCACCATCTCCTCGGCGTGGAAGAAGCCGCAGCCGCAGCC
GCAGTTACAGCAGAGGTCGTAGCTACAGCCGATCCAGGTCTCCTGTGAAGAGGGACCGTAG-
CATTGAGCGTGAAGAAAAGAGATCAAGGAGTCCTCGCCATCATAGGTCATCACCACCTCCATCAAAGGAAGGAAGCAC
AGCCTTTCACCTGATGAAAGAAGTCCAGTAGAGAGAGGTACACCATCACCAAGGGATGA-
TAGGGCAACCAATGGTTCTGACCGCAGCAGGAGCCCTAAAGATGATGTTAGAATGGATGAACGCGGAGATATTAGCCCTG
TTGAAGAAAATGGCCGTAGTCGCAGCAATAGCCCCATCCATAGGGAGGACAGAAGCCCAGTG-
GAAGACGGTAGCCCAACAGGTGACTATGAAAATCATGGTTCTCCAAGGGGTTCTCCAAGGGGCAGTGAGTCACCCCCTCT
TCCCTATATGTCGCGTTATCCTTATGATGTGCCAGATTATGCATA-
TCCATATGATGTTCCTGATTATGCATATCCATATGATGTTCCAGATTATGCTTAATGATATCCCGCGGCCATGCTAGAGTCC

GCAAAAATCACCAGTCTCTCTCTACAAATCTATCTCTCTCTATTTTTCTCCAGAA-
TAATGTGTGAGTAGTTCCCAGATAAGGGAATTAGGGTTCTTATAGGGTTTCGCTCATGTGTTGAGCATATAAGAAACCCTT
AGTATGTATTTGTATTTGTAAAATACTTCTATCAATAAAATTTCTAATTCCTAAAACCAAAATCCAGTGACCT
>pH7LIC8.1-AT2G37340 1..867 35S promoter;881..1750 AT2G37340(AthSF4Na) coding sequences; 1763..1852 3xHA tag sequence; 1866..2091 35S terminator
AGATTAGCCTTTTCAATTTCAGAAAGAATGCTAACCCACAGATGGTTAGAGAGGCTTAC-
GCAGCAGGTCTCATCAAGACGATCTACCCGAGCAATAATCTCCAGGAAATCAAATACCTTCCCAAGAAGGTTAAAGATGC
AGTCAAAAGATTCAGGACTAACTGCATCAAGAACACAGAGAAAGATATATTTCTCAA-
GATCAGAAGTACTATTCCAGTATGGACGATTCAAGGCTTGCTTCACAAACCAAGGCAAGTAATAGAGATTGGAGTCTCTA
AAAAGGTAGTTCCCACTGAATCAAAGGCCATGGAGTCAAAGATTCAAATAGAGGAC-
CTAACAGAACTCGCCGTAAAGACTGGCGAACAGTTCATACAGAGTCTCTTACGACTCAATGACAAGAAGAAAATCTTCGT
CAACATGGTGGAGCACGACACACTTGTCTACTCCAAAAATATCAAAGATACAGTCTCAGAA-
GACCAAAGGGCAATTGAGACTTTTCAACAAAGGGTAATATCCGGAAACCTCCTCGGATTCCATTGCCCAGCTATCTGTCAC
TTTATTGTGAAGATAGTGGAAAAGGAAGGTGGCTCCTACAAATGCCATCATTGCGA-
TAAAGGAAAGGCCATCGTTGAAGATGCCTCTGCCGACAGTGGTCCCAAAGATGGACCCCCACCCACGAGGAGCATCGTG
GAAAAAGAAGACGTTCCAACCACGTCTTCAAAGCAAGTGGATTGATGTGATATCTCCAC-
TGACGTAAGGGATGACGCACAATCCCACTATCCTTCGCAAGACCCTTCCTCTATATAAGGAAGTTCATTTCATTTGGAGAG
AACACGGGGGACTCTAGAGGATCCAAGGAGATATAACATTACGCCGAGGTCATGCCTCGC-
TATGATGATCGCTATGGGAACACTCGTCTTTACGTTGGCCGATTATCATCGAGAACTAGGACCCGAGACCTTGAACGTCTT
TTTAGCAGATACGGAAGAGTGCGAGATGTGGATATGAAGCGAGATTATGCTTTCGTT-
GAATTTGGTGATCCCCGTGATGCTGATGATGCAAGACATTACCTGGACGGACGAGACTTTGATGGAAGTCGCATCACTGTG
GAGTTTTCACGAGGGGCACCTCGTGGTTCTCGGGATTTTGATAGCAGAGGCCCACCACCTG-
GAGCTGGTCGCTGTTTTAACTGTGGTGTAGATGGTCATTGGGCTCGTGACTGCACAGCAGGGGACTGGAAGAACAAGTGTT
ACCGTTGTGGAGAGAGAGGACACATTGAGAGAAACTGCAAAAACAGCCCCAAGAA-
GCTTAGGCGCAGTGGAAGCTACTCCAGGTCACCTGTAAGATCCCGTTCTCCTCGTCGTAGAAGAAGCCCAAGCCGGAGTC
TTAGCCGTAGCCGAAGCTACAGCCGATCACGATCCCCGGTGAGAAGAAGAGAGAGGAGTGTG-
GAGGAGAGATCACGCAGTCCAAAGCGGATGGATGACTCTCTATCGCCAAGAGCCAGAGATCGTAGTCCGGTTCTTGATGA
TGAAGGCAGCCCAAAGATCATAGACGGGAGCCCACCACCATCACCAAA-
GCTTCAAAAGGAAGTCGGATCTGACCGTGACGGTGGTAGCCCCCAAGACAATGGCAGAAACTCTGTTGTCAGTCCTGTTG
TAGGAGCCGGTGGTGACAGTTCGAAAGAGGACCGGAGCCCTGTTGATGATGATTAC-
GAGCCAAACCGCACTTCCCCTAGAGGAAGTGAGTCTCCTCCTCTTCCCTATATGTCGCGTTATCCTTATGATGTGCCAGATT
ATGCATATCCATATGATGTTCCTGATTATGCATATCCATATGATGTTCCAGAT-
TATGCTTAATGATATCCCGCGGCCATGCTAGAGTCCGCAAAAATCACCAGTCTCTCTCTACAAATCTATCTCTCTCTATTTTT
CTCCAGAATAATGTGTGAGTAGTTCCCAGATAAGGGAATT-
AGGGTTCTTATAGGGTTTCGCTCATGTGTTGAGCATATAAGAAACCCTTAGTATGTATTTGTATTTGTAAAATACTTCTATCA
ATAAAATTTCTAATTCCTAAAACCAAAATCCAGTGACCT
>pH7LIC8.1-AT3G53500 1..867 35S promoter;881..1732 AT3G53500(AthSF4Nb) coding sequences; 1745..1834 3xHA tag sequence; 1848..2073 35S terminator
AGATTAGCCTTTTCAATTTCAGAAAGAATGCTAACCCACAGATGGTTAGAGAGGCTTAC-
GCAGCAGGTCTCATCAAGACGATCTACCCGAGCAATAATCTCCAGGAAATCAAATACCTTCCCAAGAAGGTTAAAGATGC
AGTCAAAAGATTCAGGACTAACTGCATCAAGAACACAGAGAAAGATATATTTCTCAA-
GATCAGAAGTACTATTCCAGTATGGACGATTCAAGGCTTGCTTCACAAACCAAGGCAAGTAATAGAGATTGGAGTCTCTA
AAAAGGTAGTTCCCACTGAATCAAAGGCCATGGAGTCAAAGATTCAAATAGAGGAC-
CTAACAGAACTCGCCGTAAAGACTGGCGAACAGTTCATACAGAGTCTCTTACGACTCAATGACAAGAAGAAAATCTTCGT
CAACATGGTGGAGCACGACACACTTGTCTACTCCAAAAATATCAAAGATACAGTCTCAGAA-
GACCAAAGGGCAATTGAGACTTTTCAACAAAGGGTAATATCCGGAAACCTCCTCGGATTCCATTGCCCAGCTATCTGTCAC
TTTATTGTGAAGATAGTGGAAAAGGAAGGTGGCTCCTACAAATGCCATCATTGCGA-
TAAAGGAAAGGCCATCGTTGAAGATGCCTCTGCCGACAGTGGTCCCAAAGATGGACCCCCACCCACGAGGAGCATCGTG
GAAAAAGAAGACGTTCCAACCACGTCTTCAAAGCAAGTGGATTGATGTGATATCTCCAC-
TGACGTAAGGGATGACGCACAATCCCACTATCCTTCGCAAGACCCTTCCTCTATATAAGGAAGTTCATTTCATTTGGAGAG
AACACGGGGGACTCTAGAGGATCCAAGGAGATATAACATTACGCCGAGGTCATGCCTCGC-
TATGATGATCGCTATGGAAACACTCGCCTCTATGTTGGTCGCTTATCATCTAGAACTCGTACCAGAGACCTTGAGCGTCTCT
TTAGCAGATACGGAAGAGTACGAGATGTGGATATGAAGCGTGATTATGCCTTTGTT-
GAATTTAGTGATCCTCGTGATGCTGATGACGCAAGATATTACTTGGATGGACGGGATTTCGATGGAAGTCGCATCACTGTG

GAGGCATCAAGAGGGGCTCCTCGTGGTTCGCGGGACAATGGTAGCAGAGGCCCAC-
CTCCTGGTTCTGGTCGCTGTTTTAATTGTGGTGTCGATGGCCACTGGGCCCGAGACTGCACAGCAGGAGACTGGAAGAATA
AATGTTACCGCTGTGGTGAAAGAGGACACATTGAGAGAAACTGCAAAAACAGTCCTAG-
TCCAAAGAAGGCCAGGCAGGGTGGAAGCTATTCCAGGTCACCAGTCAAATCCCGCTCCCCTCGTCGCCGAAGGAGCCCA
AGCCGTAGCCGTAGTTACAGTCGAGGTCGCAGCTACAGTCGATCGCGATCCCCAGTGAGAA-
GAGAGAAAAGCGTGGAGGACAGATCACGCAGTCCTAAGGCAATGGAGCGATCTGTATCTCCCAAAGGTAGGGACCAAA
GCCTGAGTCCAGACCGAAAAGTGATAGATGCAAGCCCAAAGCGTGGATCAGACTATGATGG-
TAGCCCAAAAGAGAATGGTAATGGCAGGAACTCTGCGAGTCCCATTGTTGGAGGTGGTGAAAGTCCTGTTGGACTTAATG
GTCAAGACAGGAGCCCGATTGATGATGAGGCTGAGCTTAGCCGTCCTTCCCCTAAAGGCAG-
TGAGTCACCTCCTCTTCCCTATATGTCGCGTTATCCTTATGATGTGCCAGATTATGCATATCCATATGATGTTCCTGATTATG
CATATCCATATGATGTTCCAGATTATGCTTAATGATATCCCGCGGCCATGCTAGAGTCCG-
CAAAAATCACCAGTCTCTCTCTACAAATCTATCTCTCTCTATTTTTCTCCAGAATAATGTGTGAGTAGTTCCCAGATAAGGG
AATTAGGGTTCTTATAGGGTTTCGCTCATGTGTTGAGCATATAAGAAACCCTTAG-
TATGTATTTGTATTTGTAAAATACTTCTATCAATAAAATTTCTAATTCCTAAAACCAAAATCCAGTGACCT

>pH7LIC8.1-LOC_Os05g02880 1..867 35S promoter;881..1849 LOC_Os05g02880(OsaSF4Na) coding sequences; 1862..1951 3xHA tag sequence; 1965..2190 35S terminator

AGATTAGCCTTTTCAATTTCAGAAAGAATGCTAACCCACAGATGGTTAGAGAGGCTTAC-
GCAGCAGGTCTCATCAAGACGATCTACCCGAGCAATAATCTCCAGGAAATCAAATACCTTCCCAAGAAGGTTAAAGATGC
AGTCAAAAGATTCAGGACTAACTGCATCAAGAACACAGAGAAAGATATATTTCTCAA-
GATCAGAAGTACTATTCCAGTATGGACGATTCAAGGCTTGCTTCACAAACCAAGGCAAGTAATAGAGATTGGAGTCTCTA
AAAAGGTAGTTCCCACTGAATCAAAGGCCATGGAGTCAAAGATTCAAATAGAGGAC-
CTAACAGAACTCGCCGTAAAGACTGGCGAACAGTTCATACAGAGTCTCTTACGACTCAATGACAAGAAGAAAATCTTCGT
CAACATGGTGGAGCACGACACACTTGTCTACTCCAAAAATATCAAAGATACAGTCTCAGAA-
GACCAAAGGGCAATTGAGACTTTTCAACAAAGGGTAATATCCGGAAACCTCCTCGGATTCCATTGCCCAGCTATCTGTCAC
TTTATTGTGAAGATAGTGGAAAAGGAAGGTGGCTCCTACAAATGCCATCATTGCGA-
TAAAGGAAAGGCCATCGTTGAAGATGCCTCTGCCGACAGTGGTCCCAAAGATGGACCCCCACCCACGAGGAGCATCGTG
GAAAAAGAAGACGTTCCAACCACGTCTTCAAAGCAAGTGGATTGATGTGATATCTCCAC-
TGACGTAAGGGATGACGCACAATCCCACTATCCTTCGCAAGACCCTTCCTCTATATAAGGAAGTTCATTTCATTTGGAGAG
AACACGGGGGACTCTAGAGGATCCAAGGAGATATAACATTACGCCGAGGTCATGCCGCGC-
TATGATGATCGTTATGGAAGCACGCGCTTGTATGTTGGTAGACTGTCTTCCCGCACTCGTTCTCGTGACCTAGAATACCATT
TCAGCAGATATGGAAGAATACGTGAAGTGGAGTTGAAGCGCGACTACGCATTTATTGAG-
TTCAGTGATCCCCGTGATGCTGAGGAAGCAAGGTACAACCTTGATGGCAGGGATGTTGATGGAAGCCGCATTCTTGTTGAG
TTTGCTAAAGGGGTTCCACGTGGTCCTGGTGGTTCACGTGAATATATGGGGAGAGGAC-
CTCCTCCTGGAACAGGTCGGTGTTTTAACTGTGGGATTGATGGTCACTGGGCAAGAGACTGCAAGGCTGGTGACTGGAAG
AACAAATGTTACCGCTGTGGGGAGAGAGGTCACATAGAAAGAAACTGTCAAAACAGCCCAA-
GAAATCTCAGGCGTGAGAGAAGTTATTCATGCTCTCCATCGCCACGCCGTGGACGTGGTCGCAGTCGGAGCTATAGCCGG
AGCCGGAGCCGGAGCCGCAGTTACAGCCGGTCTAGGTCCAGATCCCTGTCTG-
GATCTCCCAGGGCACGCAGAGAGCTTGAAAGATCAAGGAGTCTTAGTTACAGCAGGAGTCCCAGGCGATCCATCTCCCCA
GCAGCAAATGAAAAGAAGCGCAGCCCCACCCCTGATGGTAGCCGTAGTCCAAGGAG-
TCCACAGGACCAGGTGAGCCCACCACCCAAGGATAATGCTGAGCGTAATGGCTCAGATCACGGTGACAGCCCCCGGGGC
AGGGAGAACAGCAGGAGCCCGTCTGATGGCTACCGTAGCCCTGCAGCTGCCAACGGGCG-
CAGCCCAAGCCCAAGGAACAATGGAAGCCCTAGCCCAATGGACAACGGTAGCCGAAGTCCTAGGGATGGAAATGGTGA
CGGTGGTAGCCGTGGTGGCTCACGCTCACCTAGACCGAGCGAGTCTCCTGAA-
GCCCCTCTTCCCTATATGTCGCGTTATCCTTATGATGTGCCAGATTATGCATATCCATATGATGTTCCTGATTATGCATATCC
ATATGATGTTCCAGATTATGCTTAATGATATCCCGCGGCCATGCTAGAGTCCG-
CAAAAATCACCAGTCTCTCTCTACAAATCTATCTCTCTCTATTTTTCTCCAGAATAATGTGTGAGTAGTTCCCAGATAAGGG
AATTAGGGTTCTTATAGGGTTTCGCTCATGTGTTGAGCATATAAGAAACCCTTAG-
TATGTATTTGTATTTGTAAAATACTTCTATCAATAAAATTTCTAATTCCTAAAACCAAAATCCAGTGACCT

>pH7LIC8.1-LOC_Os03g17710 1..867 35S promoter;881..1870 LOC_Os03g17710(OsaSF4Nb) coding sequences; 1883..1972 3xHA tag sequence; 1986..2211 35S terminator

AGATTAGCCTTTTCAATTTCAGAAAGAATGCTAACCCACAGATGGTTAGAGAGGCTTAC-
GCAGCAGGTCTCATCAAGACGATCTACCCGAGCAATAATCTCCAGGAAATCAAATACCTTCCCAAGAAGGTTAAAGATGC
AGTCAAAAGATTCAGGACTAACTGCATCAAGAACACAGAGAAAGATATATTTCTCAA-
GATCAGAAGTACTATTCCAGTATGGACGATTCAAGGCTTGCTTCACAAACCAAGGCAAGTAATAGAGATTGGAGTCTCTA

AAAAGGTAGTTCCCACTGAATCAAAGGCCATGGAGTCAAAGATTCAAATAGAGGAC-
CTAACAGAACTCGCCGTAAAGACTGGCGAACAGTTCATACAGAGTCTCTTACGACTCAATGACAAGAAGAAAATCTTCGT
CAACATGGTGGAGCACGACACACTTGTCTACTCCAAAAATATCAAAGATACAGTCTCAGAA-
GACCAAAGGGCAATTGAGACTTTTCAACAAAGGGTAATATCCGGAAACCTCCTCGGATTCCATTGCCCAGCTATCTGTCAC
TTTATTGTGAAGATAGTGGAAAAGGAAGGTGGCTCCTACAAATGCCATCATTGCGA-
TAAAGGAAAGGCCATCGTTGAAGATGCCTCTGCCGACAGTGGTCCCAAAGATGGACCCCCACCCACGAGGAGCATCGTG
GAAAAAGAAGACGTTCCAACCACGTCTTCAAAGCAAGTGGATTGATGTGATATCTCCAC-
TGACGTAAGGGATGACGCACAATCCCACTATCCTTCGCAAGACCCTTCCTCTATATAAGGAAGTTCATTTCATTTGGAGAG
AACACGGGGGACTCTAGAGGATCCAAGGAGATATAACATTACGCCGAGGTCATGCCTCGC-
TATGATGATCATTATGGAAGCACACGCTTGTATGTTGGTAGACTGTCTTCGCGCACTCGTTCTCGTGACCTAGAATATCTTT
TCGGCAGATATGGAAGAATACGTGAAGTGGAGTTGAAGCGCGACTAC-
GCGTTTATTGAATTCAGTGATCCCCGTGATGCTGATGAAGCGCGGTATAACCTAGATGGCAGGGATGTTGATGGAAGCCGC
ATTCTTGTTGAGTTTGCTAAAGGAGTTCCACGTGGTGCTGCTGGTGGTTCAC-
GTGAATATATGGGGAGAGGACCTCCTCCAGGAACAGGTCGCTGTTTTAACTGTGGGATTGATGGTCACTGGGCAAGAGAC
TGCAAGGCTGGTGACTGGAAGAACAAATGTTACCGTTGTGGAGAAAGAGGTCACATAGAAA-
GAAACTGCCAAAACAGTCCAAGAAATCTCAGGCGAGAGAGAAGTTATTCGCGCTCCCCATCGCCACGCCGTGGACGTGG
TCATGGTCGTAGTCGTAGCTATAGCCGGAGCCGGAGCAGGAG-
TTACAGCCGATCTAGGTCCAGATCCCTATCTGGATCTCCCAGGGGACGCAGAGATCGTGATGATAGGAGATCAAGGAGTC
TTAGCTACAGCAGAAGTCCCAGGCGATCCATCTCCCCAGCAGCAAATGGAAAGGAGCG-
CAACCCCAGCCCGAATGGTAGGCGGAGTCCAAGGAGTCCACAGGATCGGGTAAGCCCACCACCCAAGGATAATGATGAG
CGCAATGGTGACAGCCCCTGGGGCAGGGAGAACAGCAGGAGCCCATCTGACGGCTACCG-
TAGCCCTGTGGCTGCCAATGGGCGCAGCCCAAGCCCAAGGAACAATGGAAGCCCTAGTCCAATGGACAACAATAGCCGA
AGCCCAAGGGACAACGGCAGCCCGAGCCCTAGGGATGGAAATGGTGATGGTGG-
TAGCCGTGGCGGCTCACGCTCGCCCAGAGCAAGCGAGTCCCCTGAAGCTCCTCTTCCCTATATGTCGCGTTATCCTTATGAT
GTGCCAGATTATGCATATCCATATGATGTTCCTGATTATGCATA-
TCCATATGATGTTCCAGATTATGCTTAATGATATCCCGCGGCCATGCTAGAGTCCGCAAAAATCACCAGTCTCTCTCTACA
AATCTATCTCTCTCTATTTTTCTCCAGAATAATGTGTGAGTAGTTCCCAGATAAGGGAATT-
AGGGTTCTTATAGGGTTTCGCTCATGTGTTGAGCATATAAGAAACCCTTAGTATGTATTTGTATTTGTAAAATACTTCTATCA
ATAAAATTTCTAATTCCTAAAACCAAAATCCAGTGACCT
>pN2tag 1..4235 Native N promoter;4236..6134 N exons I-III;6252..6269 AE18;6270..6437 TE168;6438..6509 3xFLAG tag;6570..6621 AE52;7352..8935 N exons IV-V;8936..9286 9xMyc tag;9287..9304 6xHis tag;9758..10647 N 3'-GRS
TCGAGGATCTTATTCTAATTATATGACATTTGCAACTGTGAAGGCAAGAATTTCTTACTC-
TATAATTTTTTAATTAAATATCTAATCTAAAATTTCTATAGTAAAATTGTGATTTTGTGCTCATATTCTCATATTTTTCAATGT
CTTTGTTTTTCTTTCTTGTTTTTTATTTACTTTAGGGAGGAGGGCACACAGCTCCTGAG-
TACAAACGTGAAGAGTGTTTTCATATGTTTAAAAGATGGATAACTCAACAACCTTTGTAAACATGTAACCCCATCGAAGAT
TAATTTATTAAATAGCCATTATTAAGCATCTGTCTTCTTTTCTTTCCGAT-
TTTTATGTGTGAGGGTGCAAAAATTAACTGTAAAAATAGTACGGGCTAGCCAGTTTTCGGACTAATCATTCAAAATAGTCA
ACGTTTGTCAAGTCATTGAAAAATATCCGCTATTTTGCTGCAACAGAAACCGTCCAGCATA-
TATACTGGAGTTGGGTGCACATGTGTATGTATTTCCAGTACATTATGCTGGAACTCCAACACGCGGAAAGTTCCAGCATAA
TATACTGGAGATTCGAGCACCTGTGTAAGAACTTCCAGAATATTATACTGGACCGATA-
TAGTTTGCTGGAAATCCAGTATATTATGCTGGAGTTCTAGTATATTTATGCTGGAACTCCATTATATTATCCTGGAGTTCCAG
TATACTTATGCTGGAACTCCAGTATAATATGCTGGAGTTTCACTATACTTATGCTG-
GAACTCTAGTATAATATACTGGAATATTTTCCGGATCTTGAACAATGTGTTCGTTCAAATTTATCTTTACATGAAAAGTGAC
TAAATTTTAATTACTTTTGAAAGTGTGACTATTTTTGAATGAGCACTT-
GTAAATCTGGTTATTTTTGAATTTCTCCCGAATTAACTTAGTCTAACAATATCTTGTTCTGACTGGAAAATTCAGTCTAATTA
ATTACTGCATTAACTATCTCTTCTTCTCTTTGTGAATTTTTTTTTTTTTTTTATAACAAA-
TATGAGATAATATAAAAACCTCTAGTTCTTCTTTGAAAAACAGGTGAGATTCCAATTAAAACAAAATGCCATTCTTGAACG
ATTTTGACAGGGCTTTTGCATCTATATCCACTTTTT-
GGGTCATATTTTAATTTATACCCGCTTTGCAAAAAAATTACAAGCGTATCCACTTTTCGCATAAACTTCAGGCTTACGGGTC
TGGAGTAGCAAAGGCAATCACACAAAGGTTCAGCATTCTAGGCTTTTTCGAAAACTTCAG-
CAGAATGCTGAAGTTATTTAGTTCATTTGTAAAAACTTCAGCACTAAATAAGCTGAAGTTTTGTCCTGGATTAAATAATTTT
GTCATAAAGCTTTTTCAATAACTTCAGCAGAAGATGCTGAAGTTATTTAG-
TTCATTTATAAAAACTCCAGCACTAAATAAGCTGAAGTTTTTCTTGAATTAATTAGTTTTGTCATAAAGCTTTTTCAAAAA
ACTTCAGTGCTGAAGTTATTTAGTTCGTTTTAAAATCTTCAGCAGAA-
GATGGTGAAGTTATTAAGTTCATTTGTAAAAACTTCAGCATCAGATAAGCTGAAGTTTTGTCCTGGATTCATTAGTTTTGCA

GTAAAGCTTTTTCAAAAACTTCAGCAGAAGATGCTGAAGTTATTTAGTTCATTT-
GTAAAAACTTTAGCACTAAATATGCTGAAGTTTTGCACAGGTATTAGAAAGGTGGCGCCTGAAATTGTAAAAATTAAGAT
ATATATTAAATAATTTAAAAATAAAGGTATAAATTAAATGAGAGCGATCAAA-
TAAGGCGCCTGCGCAATTTTTGATGTCAATTAGGTAGCATCAAGTTAATTTTGCACAATTTTTGCGTTTCTCTATTTAGATTG
TTTGAAAAAATGACAACTTTAATAAATTGCCGAAATAATAAAAAAAATAAACAAGTTGACAG-
TTACCTCTTTCTCCTCCCGTACAACCTTTTCACCACCACCCCTCCATGTCCATGATTTGTTGGTCCCTAAAGTTTAAATAATA
ATAAATAAATAAATAAAAAATTGTAATTAAAATTTAGAGATCAACTTT-
GGTCGTTAAATATATATTATTAAAATATTATACCGACCGAAGTTGGTCGGTATTTTATTTATCCTAAATATTTGGTTCTTTTA
ACTTAGTGACCAACGTTGGTCGCTAAATTAAAAGGACCACCAATATAGCGAC-
CAATCCATTTTGGACGCGTTTTGGTCGGTATATTGTGATAAGCGACCAACTTTGGTCGCTATTTGTGGTCTCTTTTTGCCGGA
TTTCTAGCAGTGTGTACACGCAAATCGAAAAGGATAAAATGAGATTTTTAAGGCTAACGAG-
TGCAGAATTAAATTTTAAAACGTAAGTTTAGGTCATCACATATTATGTGATTTTTAAAAAAATGATCTTCATATAGAATACA
CACGTAACACGCTTGCCCAAAAACTATTAGAACAAAATAAGTAACGGCTATTTTTAAAC-
CTTCAATCCGTAGCAGCCCACTAATCCCTGGCTCCAATTTTCTTCAATAATAAGTTGTATGCAGAAGGAAAAAGATTGTTC
CTAGAAGTTGTATGCGATACTAAACAC-
CTTCCCCCTGTTATTTTTCTGTCTGTTTTCTTTAAAGCAACGAATCCTGTGCCTTGATTCTTTTCTTGTTTCCTGTGTTAGTTAT
AAGTTTCAATAATGAAAAATAATATATTATATTGGGCGTAGGATCACAAGGGATTCAAGAA-
GCAACACTAGTCGGGAATAGATAAAGGAACATAATCAATAATCAGCATGGAAAAGGAAGAAGTAGCGAAAATTCGGCA
AGAATAATCAATTTAATTAATTACAGTAGCTAATTCTTATATATTAAGTTTCTGA-
GAAAAGTAACATTTCTTCACATTTATGGACCTACATTTGTTGTCACTTTCTATCTGCGCAAAGAAAAATAAGACCATAGTA
CTGCTTTTGGTTAGTACAACTGTTGACAAAGAAAATTACTGGGA-
TATTACCCTTCGTTTTCTTTGTAGCTTTATTTATCGGCTTGTACTTTTAGTTGTTCCTTGTGAACATATTACTGTTGAATTTGGT
GCAGGGAGGGTGGGTGGTCTTTGAAGGAATTACCTACTTCCCTTCTATTACAGTGCAAA-
GAAAACCCTATAACAATAATAATTCTAATCAACTGGAGTAAACATTAAGATGAAGCTTCACAAAAAAATCCTACAATTTA
CTTTCTATTAGGAGTAGTCGGTGGCGGATTTAGGATTTTGCGAATATGAGTGCACTATTAC-
GAAGAGGCGAATCTAGGATATAAATTTTACAGGTTTAACGTTTGGTTCTTACTATTGCACCCATTACAATTTTGAAATTATA
AGTTCAAAATTATTATTTTTTAATTGTAATTTTCTTATATCTATTTCCATACTCCG-
TACTTAAAATATTGGGATCAGTTTAACCCAATAGCATACACTGCATTATGCACTAGTTTAATATGCAAATTTTATTTAATCA
TATAAGATTTTTCGGTGACAAATAACAAATAGGAATTTTAATATGTGAAAATTTTAAAAGAA-
TAAATCAAAAAGAAAGAAAGAAAGAAAAAGAAATGTATTTAATTAATACGCACCAAGTGATGCCTAGTTTTAGAAAAGA
AAAAATAACAATAAGATTGTCATAGGAAAAAGGATTGAAAGGTCGACCAGA-
TAATTTTTTTTTTTTTTTTTTTACCAGAATGATATGTTCCACAATATATTGTACAATTTTGTCGAAACTTTATAATAACTTTCTT
AACGTTAATAAATTGGGAACAAGTTTACGATTAAATTTCACATGTGATCATTCAACTTT-
GTGTTTATTATCCAACAAAAATGAAAAATATTTTGCTAGATGAAGACTTTGTCATCCTCGGTAGAAAACTAAAATAGAAA
AAGAATTCAATCAATGGAGACCTTTTTCTCTTTGGAGCAATAATTCAATTCAATTGG-
GAAGGAATTTCCTACTCCCTTCTATTAAAGTTCAAAGAAAACCCAATAATTCCTTTTATTGCATTAAGAAGAATTTTCCTAC
TAGTGTATATCAGTTGACTAGGACACCAATAATTCTATGGAG-
TAGAGCCCATCTCACACAAACTTTTTCCAATAGCAATATAACTCTTATCTCTTCTAATATATATAAAAATTTGTTGAAAATA
TCATCTATTATTTTCTTACCACAATCACAATTTTTTCACATACAG-
TTTCTTATTCTTTTCAGAGAATTAACGTTGAGTCCATGGCATCTTCTTCTTCTTCTTCTAGATGGAGCTATGATGTTTTCTTAA
GTTTTAGAGGCGAAGATACTCGAAAAACGTTTACAAGTCACTTATACGAAGTCTTGAATGA-
TAAGGGAATAAAAACCTTTCAAGATGATAAAAGGCTAGAGTACGGCGCAACCATCCCAGGTGAACTCTGTAAAGCTATA
GAAGAGTCTCAATTTGCCATTGTTGTTTTCTCAGAGAATTATGCAACATCAAGGTGGTGTTT-
GAATGAACTAGTGAAGATCATGGAATGCAAAACTCGATTTAAGCAAACTGTTATACCGATATTCTATGATGTGGATCCATC
ACATGTTCGGAACCAAAAGGAGAGCTTTGCAAAAGCCTTTGAA-
GAACATGAAACAAAGTATAAGGATGATGTTGAGGGAATACAAAGATGGAGGATTGCTTTAAATGAAGCGGCCAATCTCA
AAGGCTCCTGTGATAATCGTGACAAGACTGATGCAGACTGTATTCGACAGATTGTTGAC-
CAAATCTCATCCAAATTATGCAAGATTTCTTTATCTTATTTGCAAAACATTGTTGGAATAGATACTCATTTAGAGAAAATAG
AATCCTTACTAGAGATAGGAATCAATGGTGTTCGGATTATGGGGATCTGGGGAATGGGGG-
GAGTCGGTAAAACAACAATAGCAAGAGCTATATTTGATACTCTTTTAGGAAGAATGGATAGTTCCTATCAATTTGATGGTG
CTTGTTTCCTTAAGGATATTAAAGAAAACAAACGTGGAATGCATTCTTT-
GCAAAATGCCCTTCTCTCTGAACTTTTAAGGGAAAAAGCTAATTACAATAATGAGGAGGATGGAAAGCACCAAATGGCTA
GTAGACTTCGTTCGAAGAAGGTCCTAATTGTGCTTGATGATATAGATAATAAA-
GATCATTATTTGGAGTATTTAGCAGGTGATCTTGATTGGTTTGGTAATGGTAGTAGAATTATTATAACAACTAGAGACAAG

CATTTGATAGAGAAGAATGATATAATATATGAGGTGACTGCAC-
TACCCGATCATGAATCCATTCAATTGTTCAAACAACATGCTTTCGGAAAAGAAGTTCCAAATGAGAATTTTGAGAAGCTTT
CATTAGAGGTAGTAAATTATGCTAAAGGCCTTCCTTTAGCCCTCAAAGTGTGGGGTTCTTT-
GCTGCATAACCTACGATTAACTGAATGGAAAAGTGCTATAGAGCACATGAAAAATAACTCTTATTCTGGAATTATTGATA
AGCTCAAAATAAGTTATGATGGATTAGAGCCCAAACAACAAGAGATGTTTTTAGATA-
TAGCATGCTTCTTGCGAGGGGAAGAAAAAGATTACATCCTACAAATCCTTGAGAGTTGTCATATTGGAGCTGAATACGGG
TTACGTATTTTAATTGACAAATCTCTTGTGTTCATCTCTGAA-
TATAATCAGGTTCAAATGCATGACTTAATACAGGATATGGGTAAATATATAGTGAATTTTCAAAAAGATCCCGGAGAACG
TAGCAGATTATGGCTCGCCAAGGAAGTCGAAGAAGTGATGAGCAACAACACAGGGACCATGG-
CAATGGAAGCAATTTGGGTTTCTTCTTATTCTAGTACTCTACGCTTTAGCAATCAGGCCGTGAAAAATATGAAAAGGCTTA
GGGTATTTAACATGGGGAGGTCGTCGACACATTATGCCATCGATTATCTGCCCAACAACTT-
GCGTTGTTTTGTTTGCACTAACTATCCTTGGGAGTCATTTCCATCTACATTTGAACTCAAAATGCTTGTTCACCTCCAACTCC
GACACAATTCTCTGCGTCATTTATGGACAGAAACAAAGGTACAATAGCTTGAATTCTATTTT-
GTTGTCATTTATTTTTCTCTCTAACTATCTTTGTCCTTTAATTTTGGTGATAATGAACAAATATTATTGTTTTTTGTTATGAAAC
AATAAAAGAAGAAGAACAATATTGCACTCGAGCAACAACAACAACAACAACCCAG-
TATAATCTCACTTAGTGGAGTCTGGGGAGGGGAGTGTGTACGCAGACCTTACCCCTATCCTGGGGTAGAGAGGCTGTTTCC
AAATAGACCCCCGACATCCTTCCCTCCAAGAACTTCGCACCTTGCTCTTGGGGAGACTGAT-
TACAAGGACGATGATGACAAGGACTACAAGGACGATGACGATAAGGACTATAAGGATGACGATGACAAGTGAACTCAC
AACCTCTTGGTTGGAAGTGGAGGTTGCTTACCATCAGAGCAACCCCTCGAGGAGAAA-
GAGGGAGATGGAATTCTTATTGAATTTTGGGGCGATTTACAATGGGGTAAGACCCCTCTATTTACAGGGGAAAAATAACTT
AGCCTCAAAATAAAGCTCTTTAAAAGATAGACATTCACTCTAAATAGAATTC-
TATTATAACACTTTTATTTAAATCATTTTAAAGTTCTTTCCTACCAAGTAAATAAGGGAAAATTTAATAACAAAAATTTAGT
TGATTTTAAAATCCTAAATATTAGAAAATTAACTTAAAATATAATTTCGTCTAGTG-
TAAAATTTATTTTTAAAGGGTAAAAAAGACGAACGACATTAAGAGCCTTTGTAATTTTAATATAGTATAAATATAAATAAT
TTACCTTTATTCAGTTTCTTAACAAGTAATTTTCCATATATAAAAAATAAATTTCTA-
TATTCACACAAAAATAATGTGTTGGCCCTCGTAATTCAAATACTATCATTCATTTCTTGTCGAGGGAGTAGTAAATACTTTT
AGGAAAGTTAGCAATAAGTAATCAAGAAATCAAGAAAACAGAGGTCATTTGATGCCCACAAA-
TACAAATGAAAAAACAAAACAAATGTTACGAAACAATAAAAGAACAAGAATAGCCTCAAAGTAAAACTCTCTGATAGA
CATTTACTCTAAATAGAATTCTATTTATAACAATCAAAAAGTTTCTACATTTATAGA-
TAGCTCCACTAGCCAAATATTTTATTATTGGAATCAGCAAAATAGGTTGTTTCTTTTTTTATTCTCATTCTGTCTGTGTTCTAA
ACAGCATTTGCCGTCTCTACGGAGGATAGATCTCAGCTGGTCTAAAAGATTGACGCGAACAC-
CAGATTTCACGGGGATGCCAAATTTGGAGTATGTGAATTTGTATCAATGTAGTAATCTTGAAGAAGTTCACCATTCCCTGG
GATGTTGCAGCAAAGTCATTGGTTTATATTTGAATGATTGTAAAAGCCTTAA-
GAGGTTTCCATGTGTTAACGTGGAATCTCTTGAATATCTGGGTCTAAGAAGTTGCGATAGTTTAGAGAAATTGCCAGAAAT
CTACGGGAGAATGAAGCCGGAGATACAGATTCACATGCAAGGCTCTGGGATAAGGGAACTAC-
CATCATCTATTTTTCAGTACAAAACTCATGTTACCAAGCTATTGTTGTGGAATATGAAAAACCTTGTAGCTCTTCCAAGCAG
CATATGTAGGTTGAAAAGTTTGGTTAGTCTGAGTGTGTCGGGTTGCTCAAAACTTGAAA-
GCTTGCCAGAAGAGATAGGGGATTTAGACAACTTACGGGTGTTTGATGCCAGTGATACTCTAATTTTACGACCTCCGTCTT
CCATCATACGCTTGAACAAACTTATAATCTTGATGTTTCGAGGCTTCAAAGATGGAGTGCAC-
TTTGAGTTCCCTCCTGTGGCTGAAGGATTACACTCATTGGAATATCTGAATCTCAGTTACTGCAATCTAATAGATGGAGGAC
TTCCGGAAGAGATTGGATCCTTATCCTCTTTGAAAAAGTTGGATCTCAGTAGAAATAATTTT-
GAGCATTTGCCTTCAAGTATAGCCCAACTTGGTGCTCTTCAATCCTTAGACTTAAAAGATTGCCAGAGGCTTACACAGCTA
CCAGAACTTCCCCCAGAATTAAATGAATTGCATGTAGATTGTCAT-
ATGGCTCTGAAATTTATCCATTATTTAGTAACAAAGAGAAAGAAACTACATAGAGTGAAACTTGATGATGCACACAATGA
TACTATGTACAATTTGTTTGCATATACCATGTTTCAGAA-
TATCTCTTCCATGAGGCATGACATCTCTGCTTCAGATTCCTTGTCACTAACAGTATTTACCGGTCAACCGTATCCTGAAAAG
ATCCCGAGTTGGTTCCACCATCAGGGTTGGGATAGTAGTGTATCAGTCAATTT-
GCCTGAAAATTGGTATATACCTGATAAATTCTTGGGATTTGCTGTATGTTACTCTCGTAGCTTAATTGACACAACAGCTCAC
TTGATTCCCGTATGTGATGACAAGATGTCGCGCATGACCCAGAAACTT-
GCCTTATCAGAATGTGATACAGAATCATCCAACTATTCAGAATGGGATATACATTTTTTCTTTGTACCTTTTGCTGGCTTATG
GGATACATCTAAGGCAAATGGAAAAACACCAAATGATTATGGGATCATTAGGC-
TATCTTTTTCTGGAGAAGAGAAGATGTATGGACTTCGTTTGTTGTATAAAGAAGGACCAGAGGTTAATGCCTTGTTACAAA
TGAGGGAAAATAGCAATGAACCAACAGAACATTCCACTGGGATAAGGAGGACTCAA-
TATAACAACAGAACTTCCTTTTATGAGCTCATCAATGGGGGTGAACAAAAGTTGATTTCTGAAGAAGATTTGAACGGTGA

ACAAAAGCTAATCTCCGAGGAAGACTTGAACGGTGAACAAAAATTAATCTCAGAAGAA-
GACTTGAACGGATCCTCTAGAGGTGAACAAAAGTTGATTTCTGAAGAAGATTTGAACGGTGAACAAAAGCTAATCTCCGA
GGAAGACTTGAACGGTGAACAAAAATTAATCTCAGAAGAAGACTTGAACGGATCCTCTA-
GAGGTGAACAAAAGTTGATTTCTGAAGAAGATTTGAACGGTGAACAAAAGCTAATCTCCGAGGAAGACTTGAACGGTGA
ACAAAAATTAATCTCAGAAGAAGACTTGAACGGACATCACCATCACCATCACTGATGTACAT-
ATCAACAACGAGTTTTTAAAGGATTCCAACAAGTATAACTTTTTATGCTCAAATCAGCTCCTTGTATTGTGGAGAAAGCTG
AGTACGAGATGAAGTTGACGTCCGTTATCCTTTATGATCTCTCTGTTCTTTGTGTTAACTT-
GCCTACTTCATCAGATGAATAACAGAAGCCCGTTCCTCTCATTCTCAACACTGTTTGCACGTCTGTTGTTACTTGTTAAAAT
GGATCTTGATAAAGTAATAACATCTCTATATTACTTATAAGTGGTTTTAACAAGTTCAC-
TCTTTTGCTTTTGCAGTTCAAATGGGAACACAATGTATATTGAGAACTAGAACAATGACACTGCATATATATATATATATGT
ATGTATGTAATTCTCGTCTTTTGGACTAGAATACCTT-
GTTTCATTATGAAATGAATTAACATCTTCGCCTTTGCTGACAAGTAACCAATTACAGATGAATGAAATCACCTGATCAACA
TTCATTAGCTTTGTATTCTTTGACGATTTCGGTTTCATAACTCTTTCCCCTGCAGTTAAAA-
TATGTAGTTAGCCCGATTGCACCTCTAGGGCGCAGCGGAGTATTAAAAAAAAAAAGATCTTTCTCATTTGTCTAAGTCTTG
GTAGTCAGAATTACGAGTTTGTATAAAGTTGGCTCAAACATCACCTTTGTATAA-
GAAAAATACATACACACACAGTAGAAAGAAACAGATACCTTCGCAAATTTGATTGGGAGGTACTGATTTCTTCTTTCAGTT
GGCGATTAGCCTCTTGTGTCATCTTTGGAGCTTCTTATGAT-
TTTTTTTTCTTAGGTAAAATTCATTTAATAATTTGTTAATCATATTACTGTTGGGCTAAACTAGCCCCGATACACTCATAACA
TGGTGTGATATTGTTCGCTTTGGGCCAAGCCCGTATGGTTTTCCCCAAAAGGCCTCGCAC-
CATTAAGAGATCCATACACCTTAAATGTAGACTCACAATCTTTTCAGCTATTAATGTGGCACTTTATTCGCATACCCAACAT
TATGTCTACACTACAGGAATTAGAGTTGGAACAGAGTTTTAAAACTAGTCAAAGAGTTTT-
GGAGCTAACAAAACTATCTTGATAAATATAATACAAACAACTCGTAGTGTTCAGAGGCGGAATAACTATGTGATTACTGT
AGAAACTTATAAACTTTAAATTTTGGATTCGCATTTGCTTACCGTTGATTTTC-
TATCTCATTTATCTTGGCTGGTTGTGCCATAATTAAATCCATTGGAGGGACATTGTAGGATTAGCTTACGTAAATGTGCTTG
TAAATTGAATAACGTGAGCTAACATTGTTGAC

Data S2. SR family protein sequences from Tomato, tobacco, Arabidopsis, and rice.

>AT1G09140
MSSRWNRTIYVGNLPGDIRKCEVEDLFYKYGPIVDIDLKIPPRPPGYAFVEFEDPRDADDAI-
YGRDGYDFDGCRLRVEIAHGGRRFSPSVDRYSSSYSASRAPSRRSDYRVLVTGLPPSASWQDLKDHMRKAGDVCFSEVFPDRKGM
SGVVDYSNYDDMKYAIRKLDATEFRNAFSSAYIRVREYESRSVSRSPDDSKSYRSRSRSRGP-
SCSYSSKSRSVSPARSISPRSRPLSRSRSLYSSVSRSQSRSKSRSRSRSNSPVSPVISG
>AT1G02840
MSSRSSRTVYVGNLPGDIREREVEDLFSKYGPVVQIDLKVPPRPPGYAFVEFDDAR-
DAEDAIHGRDGYDFDGHRLRVELAHGGRRSSDDTRGSFNGGGRGGGRGRGDGGSRGPSRRSEFRVLVTGLPSSASWQDLKDHM
RKGGDVCFSQVYRDARGTTGVVDYTCYEDMKYALKKLDDTEFRNAFSNGYVRVREYDSRK-
DSRSPSRGRSYSKSRSRSRGRSVSRSRSRSRSRSRSPKAKSSRRSPAKSTSRSPGPRSKSRSPSPRRSRSRSRSPLPSVQKEGSKSPSKPSPA
KSPIHTRSPSR
>AT1G23860
MTRVYVGNLDPRVTERELEDEFKAFGVLRNVWVARRPPGYAFLEFDDERDAL-
DAISALDRKNGWRVELSHKDKGGRGGGGGRRGGIEDSKCYECGELGHFARECRRGRGSVRRRSPSPRRRRSPDYGYARRSISPRGR
RSPPRRRSVTPPRRGRSYSRSPPYRGSRRDSPRRRDSPYGRRSPYANGV
>AT1G55310
MRGRSYTPSPPRGYGRRGRSPSPRGRYGGRSRDLPTSLLVRNLRHDCRQEDLRKS-
FEQFGPVKDIYLPRDYYTGSHLCTDTCKASRDPRGFGFVQFMDPADAADAKHHMDGYLLLGRELTVVFAEENRKKPTEMRARER
GGGSRFRDRRRTPPRYYSRSRSPPPRRGRSRSRSGDYYSPPPRRHHPRSISPREE-
RYDGRRSYSRSPASDGSRGRSLTPVRGKSRSLSPSPRRSISRSPRRSRSPSPKRNRSVSPRRSISRSPRRSRSPRRSRRSYTPEPARSRSQSP
HGGQYDEDRSPSQ
>AT2G37340 AthSF4Na
MPRYDDRYGNTRLYVGRLSSRTRTRDLERLFSRYGRVRDVDMKRDYAFVEFGDPRDAD-
DARHYLDGRDFDGSRITVEFSRGAPRGSRDFDSRGPPPGAGRCFNCGVDGHWARDCTAGDWKNKCYRCGERGHIERNCKNSPK
KLRRSGSYSRSPVRSRSPRRRRSPSRSLSRSRSYSRSRSPVRRRERSVEERSRSPKRMDDSL-
SPRARDRSPVLDDEGSPKIIDGSPPPSPKLQKEVGSDRDGGSPQDNGRNSVVSPVVGAGGDSSKEDRSPVDDDYEPNRTSPRGSESP

>AT2G46610
MRHVYVGNFDYDTRHSDLERLFSKFGRVKRVDMKSGYAFVYFEDER-
DAEDAIRRTDNTTFGYGRRKLSVEWAKDFQGERGKPRDGKAVSNQRPTKTLFVINFDPIRTRERDMERHFEPYGKVLNVRMRRN
FAFVQFATQEDATKALDSTHNSKLLDKVVSVEYALREAGEREDRYAGSRRRRSPSPVYRRRP-
SPDYTRRRSPEYDRYKGPAPYERRKSPDYGRRSSDYGRARARSPGYDRSRSPIQRARG
>AT2G24590
MSRVYVGNLDPRVTERELEDEFRSFGVIRSVWVARRPPGYAFLDFEDSRDARDAI-
REVDGKNGWRVEQSHNRGGGGGRGGGRGGGDGGRGRGGSDLKCYECGESGHFARECRSRGGSGGRRRSRSRSRSPPRYRKSPT
YGGRRSYSPRARSPPPPRRRSPSPRGRNYSRSPPPYRARDEVPYANGNGLKDVRRSRS
>AT3G55460
MRRYSPPYYSPPRRGYGGRGRSPPPPPPRRGYGGGGGGGGRRGSSHGSLLVRNIPLD-
CRPEELREPFERFGPVRDVYIPRDYYSGQPRGFAFVEFVDAYDAGEAQRSMNRRSFAGREITVVVASESRKRPEEMRVKTRTRSREP
SGSRDRSHGRSRSRSISRSRSPRRPSDSRSRYRSRSYSPAPRRRGGPPRGEEDEN-
YSRRSYSPGYEGAAAAAPDRDRNGDNEIREKPGYEAEDRRRGGRAVSRSPSGSRSRSVEVSPR
>AT3G49430
MSGRFSRSIYVGNLPGDIREHEIEDIFYKYGRIVDIELKVP-
PRPPCYCFVEFEHSRDAEDAIKGRDGYNLDGCRLRVELAHGGRGQSSSDRRGGYGGGGSGYGGGGGGGGSARFGVSRHSEFRVI
VRGLPSSASWQDLKDHMRKAGDVCFAEVTRDSDGTYGVVDYTNYDDMKYAIRKLDDTEFRN-
PWARGFIRVKKYESSRSRSRSPSRSRSRSRSRSRGRGRSHSRSRSLSRSKSPRKDLSKSPRRSLSRSISKSRSPSPDKKKSPPRAMSRSK
SRSRSRSRSPSKSPPKVREGSV
>AT3G53500 AthSF4Nb
MPRYDDRYGNTRLYVGRLSSRTRTRDLERLFSRYGRVRDVDMKRDYAFVEFSDPRDAD-
DARYYLDGRDFDGSRITVEASRGAPRGSRDNGSRGPPPGSGRCFNCGVDGHWARDCTAGDWKNKCYRCGERGHIERNCKNSPS
PKKARQGGSYSRSPVKSRSPRRRRSPSRSRSYSRGRSYSRSRSPVRREKSVEDRSRSPKAMERSVSPKGRDQSLSPDRKVIDASPKRGS
DYDGSPKENGNGRNSASPIVGGGESPVGLNGQDRSPIDDEAELSRPSPKGSESP
>AT3G61860
MRPVFVGNFEYETRQSDLERLFDKYGRVDRVDMKSGYAFVYFEDERDAEDAIRKLD-
NFPFGYEKRRLSVEWAKGERGRPRGDAKAPSNLKPTKTLFVINFDPIRTKEHDIEKHFEPYGKVTNVRIRRNFSFVQFETQEDATKA
LEATQRSKILDRVVSVEYALKDDDERDDRNGGRSPRRSLSPVYRRRPSPDYGRRP-
SPGQGRRPSPDYGRARSPEYDRYKGPAAYERRRSPDYGRRSSDYGRQRSPGYDRYRSRSPVPRGRP
>AT3G13570
MRGRSYTPSPPRGYGRRGRSPSPRGRFGGSRDSDLPTSLLVRNLRHD-
CRQEDLRRPFEQFGPVKDIYLPRDYYTGDPRGFGFIQFMDPADAAEAKHQMDGYLLLGRELTVVFAEENRKKPTEMRTRDRGGR
SNRFQDRRRSPPRYSRSPPPRRGRRSRSRSRGYNSPPAKRHQSRSVSPQDRRYEKER-
SYSRSPPHNGSRVRSGSPGRVKSHSRSPRRSVSPRKNRSYTPEQARSQSPVPRQSRSPTPVPRGAQNGDRSPSQ
>AT4G31580
MSRVYVGNLDPRVTERELEDEFRAFGVVRSVWVARRPPGYAFLDFEDPRDARDAIRALD-
GKNGWRVEQSHNRGERGGGRGGDRGGGGGGRGGRGGSDLKCYECGETGHFARECRNRGGTGRRRSKSRSRTPPRYRRSPSYG
RRSYSPRARSPPPPRRRSPSPPPARGRSYSRSPPPYRAREEVPYANGNGLKERRRSRS
>AT4G25500
MKPVFCGNFEYDAREGDLERLFRKYGKVERVDMKAGFAFVYMEDER-
DAEDAIRALDRFEFGRKGRRLRVEWTKSERGGDKRSGGGSRRSSSMRPSKTLFVINFDADNTRTRDLEKHFEPYGKIVNVRIRRNF
AFIQYEAQEDATRALDASNNSKLMDKVISVEYAVKDDDARGNGHSPERRRDRSPER-
RRRSPSPYKRERGSPDYGRGASPVAAYRKERTSPDYGRRRSPSPYKKSRRGSPEYGRDRRGNDSPRRRERVASPTKYSRSPNNKRER
MSPNHSPFKKESPRNGVGEVESPIERRERSRSSPENGQVESPGSIGRRDSDGGYDGAESPMQKSRSPRSPPADE
>AT4G02430
MSSRSSRTIYVGNLPGDIREREVEDLFSKYGPVVQIDLKIPPRPPGYAFVEFEDARDADDAI-
YGRDGYDFDGHHLRVELAHGGRRSSHDARGSYSGRGRGGRGGGDGGGRERGPSRRSEYRVVVSGLPSSASWQDLKDHMRKGG
EVCFSQVFRDGRGTTGIVDYTSYEDMKYALDDTEFRNAFSHEY-
VRVREYDSRRDSRSPSRGRSYSKSRSRGRSPSRSRSRSRSRSKSRSPKAKSLRRSPAKSTSRSPRSRSRSKSRSLSPRGWVTVERHWIALI
>AT5G18810
MARARSRSRSYSPRPRDRSPPRERKGYDDNRLRERPSSRDHESSGPSGLLIRNLPLDARP-
NDLRDSFERFGPLKDIYLPRNYYTGEPRGFGFVKYRYAEDAAEAMKRMNHKVIGGREIAIVFAEENRKTPQEMRTTNGTSGRHGD
YKRTSHRSPRRRYRSHSRSRSPPRRESRHSKVREDDLYSPRRRSRSISRSPLPRNEREYKSRNCRSPREERVLTPIRSRCLSRSRSRSLSR
>AT5G52040

MKPVFCGNFEYDARESDLERLFRKYGKVERVDMKAGFAFVYMEDER-
DAEDAIRALDRFEYGRTGRRLRVEWTKNDRGGAGRSGGSRRSSSGLRPSKTLFVINFDAQNTRTRDLERHFEPYGKIVNVRIRRNF
AFIQYEAQEDATRALDATNSSKLMDKVISVEYAVKDDDSRGNGYSPER-
RRDRSPDRRRRSPSPYRRERGSPDYGRGASPVAHKRERTSPDYGRGRRSPSPYKRARLSPDYKRDDRRRERVASPENGAVRNRSPR
KGRGESRSPPPYEKRRESRSPPPYEKRRESRSPPPYEKRRERSRSRSKSSPENGQVESPGQI-
MEVEAGRGYDGADSPIRESSPSRSPPAEE
>AT5G64200
MSHFGRSGPPDISDTYSLLVLNITFRTTADDLYPLFAKYGKVVDVFIPRDRRTGDSRG-
FAFVRYKYKDEAHKAVERLDGRVVDGREITVQFAKYGPNAEKISKGRVVEPPPKSRRSRSRSPRRSRSPRRSRSPPRRRSPRRSRSPR
RRSRDDYREKDYRKRSRSRSYDRRERHEEKDRDHRRRTRSRSAS-
PDEKRRVRGRYDNESRSHSRSLSASPARRSPRSSSPQKTSPAREVSPDKRSNERSPSPRRSLSPRSPALQKASPSKEMSPERRSNERSPS
PGSPAPLRKVDAASRSQSPYAAE
>LOC_Os01g06290
MPRYDDRDRYDDRYGGNTRLYVGRLSSRTRTRDLEDLFGRYGRVRYVDMKHEFAFVEFSDAR-
DADEARYNLDGRDFDGSRMIVEFAKGVPRGPGGSREYMGRGPPPGSGRCFNCGIDGHWARDCKAGDWKNRCYRCGDRGHIER
DCRNSPKNLKRGRSYSRSPSPRRGRSRGRSYSRSRSRSYSRSQSPRRDSRN-
ERRSRSPRDSRSPRGSPRDSRSPRGSPRDSRSPKGSPRDTQSPRGSPRDSRSPRRSASPPNGRNRSPTPNASRSPAPRDSRSPMRADSRS
PADHERRDMSTAANGRSPSPRDYEDNGNHRASPRGSASP
>LOC_Os01g21420
MAIRKFMYENIQSLHKTSEAITKHCLDLPFLTATGFGISPMFHKDIDLAGSVGSWSKI-
HYEVAKCKSARTQNACTAGIYTYLAETMSRRNSRTIYVGNLPGDIREREVEDLFYKFEDPRDADDAICGRDGYNFDGYRLRVELA
HGGRGQSYSYDRPRSYSSGRRGGVSRRSEYRVMVTGLPSSASWQDLKDHMRRAGDVCFSDVY-
REAGATVGIVDYTTYEDMKYAIRKLDDSEFRNAFSRAYIRVWNLFLGHRHLWMKDQYQDHGLLFLHLLVEDLLVKAQAGAYPA
LPLR
>LOC_Os02g03040
MRPVFCGNLDYDARQSEIERLFSKYGRVERVDMKSGFAFVYMEDERDADEAIHRLD-
RIEFGRKGRRLRVEWTKEDRSGGRRGNSKRSPNNTRPTKTLFVINFDPINTRTRDLERHFDQYGKISNVRIRRNFAFVQYELQEDAT
KALEGTNGSTLMDRVISVEYALRDDDEKRNGYSPERRGRDRSPDRRDYR-
GRSASPYGRGRERGSPDYGRGRERGSPDYGRGGDRGSPDYHRGASPQGGNKGDERGSPPNNYDRERREASPGYDRPRSRSPARYE
RE
>LOC_Os02g15310
MRRYSPPYRSPPRRGYGGRGRSPPRRGYGGRKEQGSGSLLVR-
NIPLSCRAEDLRVPFERFGPVRDVYLPKDYYTGEPRGFAFVEFVDPYDASEAQYHMNRQVVFGREITVVLAAESRKRPEEMRSRAR
VRGYSDHEGRRSSHYGRSRSRSRSRSPRYRGRPRSRSYSPAPRRRDDYSASPQRK-
DTHRAKSPRRQPKEHEVDKKRRSYSPANKDGDRRDADNGYEKRSPPADSDGSPPHRRSPRQSSGSPPGSRSRSADGSPARSD
>LOC_Os02g39720
MARVYYVGNLDPRVTAREIEDEFRVFGVLRSVWVARKPPGFAFIDFDDRRDAEDAIRDLD-
GKNGWRVELSTKAGSGRGRDRSGGSDMKCYECGEPGHFARECRLRIGSGGLGSGRRRSRSRSRSPRYRGRSRSRSPRYRRSPSYGRR
SYSPRDRSPKRRSYSRSPPPARARSYSRSPPPPRERSYSRSPAQPANREESPYANNA
>LOC_Os02g54770
MARLYVGNLDPRVTSGELEDEFRVFGVLRSVWVARKPPGFAFIDFDDKRDAEDALRDLD-
GKNGWRVELSRNSSSRGGRDRHGGSEMKCYECGETGHFARECRLRIGPGGLGSGKRRSRSRSRSRSPQYRKSPTYGRRSYSPRDRSP
RRRSVSPVRGRSYSRSPRGRGGSPYADGRDGGRYRRSRS
>LOC_Os03g17710 OsaSF4Nb
MPRYDDHYGSTRLYVGRLSSRTRSRDLEYLFGRYGRIREVELKRDYA-
FIEFSDPRDADEARYNLDGRDVDGSRILVEFAKGVSSSLVPRGAAGGSREYMGRGPPPGTGRCFNCGIDGHWARDCKAGDWKN
KCYRCGERGHIERNCQNSPRNLR-
RERSYSRSPSPRRGRGHGRSRSYSRSRSRSYSRSRSRSLSGSPRGRRDRDDRRSRSLSYSRSPRRSISPAANGKERNPSPNGRRSPRSPQD
RVSPPPKDNDERNGDSPWGRENSRSPSDGYRSPVAANGRSPSPRNNGSPSPMDNNSRSPRD-
NGSPSPRDGNGDGGSRGGSRSPRASESPEA
>LOC_Os03g22380
MSRRWSRTIYVGNLPGDIREREVEDLFYKYGRIVDIDLKIPPRPPGYAFVEFEDPRDAEEA-
CAGRDGYNFDGHRLRVEPAHGGRGNGGSSFDRPSNFGGGGRRGVSRHSEYRVLVTGLPSSASWQDLKDHMRKAGDVCFSEVYR

EGGGTVGIVDYTNYDDMKYAIKKLDDSEFRNAF-
SKGHIRVKEYDGKRARSYSRSRSPSRSRSKSRSLSKSPRTRRSASRSRSRSRSVSSRSRSASKGRSPSRSPARSKSPNASPANGEASSPKK
RSPSRSPSRSRSPDAKSE
>LOC_Os03g24890
MAGYRSRSRSYSPQPRRRYSRSPPRYKRYDDPRDRYPRGGGGGGGGEGPRRGYGRP-
PAPTGLLVRNISLTARPEDIRIPFEQFGPVKDVYLPRNFHTRELRGFGFVKFRYPEDAAVAKQELNHQVIGGREISIVFAEENRKTPQ
EMRMRTRTSGRYMDGSHRRRSVSRSPRSRY-
HSYSPSPSPARRDYRDHRDDYSPGESLSPHGQDKRHHRSNGRSASPDELERHVSPSNNGHGPPVDGKS
>LOC_Os03g25770
MGRGYDYGPSPPREYRRRARSPSPRGRYGGRDRDLPTSLLVRNLRRD-
CRPDDLRRPFGKFGRVKDIYLPRDYYTGEPRGFGFIQYYDPEDAADAKYHMDGQILLGREVTVVFAEENRKKPSEMRARERVGSR
DRSYDRRSRSPRYSRSRSPVYSPRSRSRSRSYSPAPKRKHYSSRSPARRERSLSRSPADSRSRSRSLSDDRRSKSPDRERSLSVSR
>LOC_Os03g27030
MSRLGRYGPPAVRDSYSLLVLNVSFRTTADDLLALFDRCGEVLDIRIPRDRRTGDSRG-
FAFVRYKYEDEAQKAVDKLDGRTVDGRDIMVQFAKYGPNAERIHKGRIVEEISKPRGRSRSPSPKRRYRADYRDRGYRDDYRDRH
YRDDYQDREYRRSRSIERYERERYQEKGYRRRSRSISPDYDRRHRKNGRPRDERSPRSRSPLT
>LOC_Os04g02870
MRPVFVGNLDYDTRHSELDRLFYRYGRIDRIDMKSGFAFVYFEDERDGDEAIRALD-
GYPFGPGRRRLSVEWSRGDRGSRRDGYSKPPVNTKPTKTLFVINFDPINTRVTDIERHFEPFGKLSNVRIRRNFAFVQFETQEEATKA
LEATHSTKLLDRVISVEYAFRDDTERGDRYDGARGGYGRRDDSPYRRSVSPVYRSRPSPDY-
GRQRSPVYGSYDRSPVNDRYRSRSPVRRSRSPLANRRAYD
>LOC_Os05g02880 OsaSF4Na
MPRYDDRYGSTRLYVGRLSSRTRSRDLEYHFSRYGRIREVELKRDYA-
FIEFSDPRDAEEARYNLDGRDVDGSRILVEFAKGVPRGPGGSREYMGRGPPPGTGRCFNCGIDGHWARDCKAGDWKNKCYRCG
ERGHIERNCQNSPRNLR-
RERSYSCSPSPRRGRGRSRSYSRSRSRSRSYSRSRSRSLSGSPRARRELERSRSLSYSRSPRRSISPAANEKKRSPTPDGSRSPRSPQDQVSP
PPKDNAERNGSDHGDSPRGRENSRSPSDGYRSPAAANGRSPSPRNNGSPSPMDNGSRSPRDGNGDGGSRGGSRSPRPSESPEA
>LOC_Os05g07000
MSRYVDPNDRYEPRGRNSRLYVGHISLRTRAEDLENLFSRYGRVRFVDLKNEYGFVEFSDPR-
DANDARLDLDGRKYDGSDIIVQFARGVERGLGGSRGYKARPAHGSDHCFNCGMEGHWHRNCTAGDWTNRCYGCGERGHILR
ECKNSPKDLKQERGYSRSRSPRRRRSPSYGKSGPPSHWGSHGADREERLHSRRDGRGY-
SRSPRRHDSPSNQRNHSPRRYALPSNERYDGTRRYASPSYGRDRSPGGNALPANGRSHNLTSDGMNPSPRERDDQNGSHRRGDN
DYLPSKRDDQNGSYRRGDSDYLSRERDDYLSRGRDDQNGSHRRGDSDYLSRDR
>LOC_Os05g30140
MSRRNSRTIYVGNLPGDIREREVEDLFYKYGRIVDIDLKIPPRPPGYAFVEFEDPRDAQDAI-
YGRHGYDFDGHRLRVELAHGGRGPSFDRSSSYSSAGRRGAAKRTDYRVMVTGLPSSASWQDLKDHMRRAGDVCFSDVYREGGA
TIGIVDYTNYEDMKQAIRK-
LDDSEFRNAFSRAYIRVREYDSAKRSRSRSRGRSHSRSISRSRSRSRSISRSRSRSYSRSRSPRSRSASRSHSPVKERSRSASQSRSPVKERS
PSRSPPPATSPPREKSASKSPVKSRSLSRSPSPVKSE
>LOC_Os06g08840
MARVYYVGNLDPRVTARELEDEFRVFGVLRSVWVARKPPGFAFIDFDDRRDAQDAIRDID-
GKNGWRVELSRNASSGRGGRDRYGSSESKCYECGETGHFARECRLRIGSGGLGSGRRRSRSRSRSRSPRYRRSPSYGRRSYSPAGRSP
RRRSVSPARARSYSRSPQYNRGRDESPAYDNGYRRSRS
>LOC_Os07g43050
MSRFGRSGPPPIRDTYSLLVLNITFRTTADDLSPLFEKYGEVVDIYIPRDRRTGDSRG-
FAFVRYKYEDEAQKAVDRLDGRVVDGREIMVQFAKYGPNAERIHKGKIMETVPRSRGRSRSRSPRRGYRDDYRERDYRKRSRSRD
RYGRDRYRERDYRRRSRSRSYTPDDYRRRGRDSVSPARRSLSRSRSRSYSPD-
DYRKRGKHSQSPVCKSPSAKRSPSMSPARRSPSASPARRSPSASPVRRSPSRSPRRTPSSQEGSPVKRYDEPRRSRSPST
>LOC_Os07g43950
MGRGYSYSPSPPPRSYRRRASSPIPRDRYGGRGRDLPTSLLVRNLRRD-
CRPEDLRRPFGQFGRLKDIYIPRDYYSGEPRGFGFVQYYDPDDAADAKYYMDGQVILGREVAVVFAEENRKKPSEMRSRDRISGSR
GRSYDQRYSRSPRYSPPPRGRSPYRSPSYSRSPSPRYARRRMRERSYSPVESRSRSRSPVEEGYGGGSTRRERSLSVSE
>LOC_Os07g47630
MSRRWSRTIYVGNLPGDIREREVEDLFYKYGRIVDIDLKIPPRPPG-
FAFVEFEDPRDAEDAIRGRDGYNFDGNRLRVELAHGGRGNSSSFNNSGGGGRRGGVSRHTEYRVLVTGLPSSASWQDLKDHMR

NAGDVCYSEVYREGGGTIGIVDYTNYDDMKYAIRKLDDSEFKNAF-
SKAYIRVKEYDGKRSRSYSRSRSRSRGRSYSRSRSPSKSPKGKSSRRSASRSRSRSASSRSRSESKGRSPSRSPARSQSPNTSPANGDAAS
PKKRSPSRSPPKKRSPSRSPSRSRSPDAKSE
>LOC_Os08g37960
MSHFGRSGPPDIRDTFSLLVLNISFRTTADDLFPLFDRYGKVVDVFIPRDRRTGDSRG-
FAFVRYKYAEEAQKAIDRLDGRNVDGRNIMVQFAKYGPNAEPIRKGRIIEEVEKSRDRSRSRSPRRRHRDDHRDRERRGRSRSRSRE
RHGRDRDRDHRRHSRSRSRSRSRSRSRSLSPDYKNRRRGRDDDKRRSKSKSKSKSRSKSRSRSKSRSKSRSRSRSRSRSRSFHSASPTR
HSASPPLKGSTPRRSPARNGSPEKETNGKGSPPSRSVSPSPQRAGSRSPGSDDKE
>LOC_Os11g47830
MRSRSPSKRRRHGSRGRSPPSSRHGCAKDKE-
GAAVSLFVSNLPRSCRPEDVQVPFQKFGPVRDVYLPKDYNTGEPRGFAFVEFAHSSDASKARYHMNRKMLSGREISVAFAVQTRK
RPEEMRRIIGARHNSPQRKEECRTNSPGQPKGH-
DEKRKRRSYTPKYKDRQYADIGRDETPPAPDSERPWALCRSPRPSPPGQSHSRSYSRSHSLHLHDHARTRSCSPAPGRQDDQYASP
QRKEHQTKSSGQTKGHDDMRRSYTPEYNECQDADNGFDETPPAPDGERSSVLGRSPRP-
SPPGRSHCHSHSRSRSPELRGHARSRSCSPATGRQDNQSTSPQRREKHQTKSSGQAKEHDEKRRSYTPEYNDRRDADNGYDQTPP
APDGERSWALGRSPQPSPPGRSHFHSHSRSRSPELRGRARSRSCSPAPGRQGDDQYASPQR-
KEEQQTKSSRQTKEHDEERRSCTPEYSDRRDAFIGHDETPPSAEWGSKLGTVQVISNTAASSN
>LOC_Os12g38430
MRRYSPPYRSPPRRGYGGRGRSPPRRGYGGRREQGSGSLLVR-
NIPLSCRGEDLRVPFERFGPVRDVYLPKDYYSGEPRGFAFVEFVDPYDASEAQYHMNRQVFFGREITVVLAAESRKRPEEMRSRAR
VRGYSGNEGRRSSYYGRSRSRSRSPHYRGRPRSRSYSPAPRRRDDYSASPPRKDTHPTK-
SPRRQPKEHDEEKKRRSYSPASRDGDPRDADNGYEKRSPPPDSDGSPPHRRSPRHSSGSPPGSRSRSADVSPARSD
>Solyc01g005820.4.1
MRRRSYSPSPPRGYGRRGRSPSPRGRYAGHGRDGPTSLLVRNLRHD-
CRPEDLRRPFGQFGPVKDIYLPKDYYTGEPRGFGFVQFVDPADAADAKYQMDGQGFQGRQLTVVFAEENRKKPTEMRSRERSGS
HRSSRSYDRRRTPPSRYARPGSHSRDYSPKRRPYSRSVSPEEKRYSRERSYSRSPPRDLSPP-
PHNGSRSRSQTPVREHPPYNGSPRSRSRSPVRRERSPVRGHSRSPSRSRSRSPGCAPYSP
>Solyc01g080660.3.1
MGRYRSRSRSLSRSYSPVRRKRHDEPRDRRRERRSPGPSGLLVR-
NIPLSARPEDLRVPFERYGPIRDVYLPKNYHTGEPRGFGFVKFRYAEDAAEAKAHLNNTVIGGRDIRIVFAEDNRKTPREMRKVLS
TSGPSARGSYWRHSSPSRRYHSYSRSASPARRDSRC
>Solyc01g091750.3.1
MRAIFCGNLEFDARQSDVERLFRRYGKVDRVDMKSGFAFIYMEDERDADDAIRRLD-
RIEFGKKGRRLRVEWTKDRGSRRPEISRKPAANTRPSKTLFVINFDPVHTQTRDIEKYFEPYGRISNVRIRKNFAFVQYESVDDASRA
LEATNMSKFMDRVISVEFAIRDDDDRRNGRSPDRRGRDMSPDRRGYDRRRSPSPYRRDRG-
SPDYGRGAPLNSRPQTRRSPEYGRAESPVNERYHSRSPPPRERSRS
>Solyc01g096180.4.1
MRPLFVGNIEYDIRQPELERLFSKYGRIERLDMKSGLHILAGFAFVYFEDERDAADAIRCLD-
NMPFGYDKRRLSVEWAKGDRVQPRDDSKVSANQRPTRSLFVINFDPIRTRVRDIERHFEPYGKILNVRIRRNFAFVQFENLEDASK
ALECTHMSEILDRVVSVEYALRDDGERGDRYDSPRRDYIRHGDSPYRRSPSPMYRRGRP-
SPDYGRPGIPAYDKYNGSSYDRYRSPEYGSYRRFPVRR
>Solyc01g099810.3.1
MSYSNMGRLSRTIYVGNLPGDIREREVEDLFYKYGPIVEIDLKVP-
PRPPGYAFVEFEDPRDADDAIRGRDGYDFDGHRLRVELAHGGRGSSSYDRHSSYSSASRSGLSRRSDYRVLVSGLPSSASWQDLKD
HMRRAGDVCFSQVFRDRDGMRGIVDYTNYDDMRYAIK-
KLDDSLFRNQFSRAYIRVDKYDKRHSYSRSPSPYNSRSRSYSRSRSPRRSYSSQSGSVSPRGKYSRRSVSISPSRAFSPALSLSRSGFRGD
LGIAI
>Solyc01g105140.3.1
MSHFGRTGPPDIADTYSLLVLNITFRTSADDLFPLFDKYGKVVDIFIPRDRRTGESRG-
FAFVRYKYAEEAQKAVDRLDGRVVDGREMAVQFAKYGPNAERIHQGRIIEKVPGFKGSSRSRSPRRRYRDDYHRDREYRRSRSRS
VDRYERDRYRQRERDYRHRSRSRSLSPDYDRDRGRRRDRKHYRRSPSVD-
SASPSRRSPSPHRKESPPRSLSPTKGSPVRRVRNERSPTPRSRSPPGRAMDSRSPSPRVDED
>Solyc03g026240.4.1
MRPIFCGNFEFETRQPELERLFKRYGKVDRVDMKSGFAFVYMDDERDAKDAIQGLD-
RIEFGRKGRRLRVEWSKEERSRKPEGSKKSSSSFRVSKTLFVINFDPYNTRTRDLERHFDPYGKILNIRIRRNFGFIQFETQEDATRALD

ATNMSKLMDRVITVEYAIRDDDDRKNGYGPGKTYNQSPRRGYDRGRSRSPRGRDRLSPDY-
GRGRDRPSPDYGRGRDRPSPDYGRGRDRPISDFDRGRDRPNSDFGRGRDQLSPDYGRGPSRSPKHREGNSEYGRGHSPAVGKERN
PGHGNVRSPSPRRERTGPGNGLMSSPLNISPGYGDGPSPSAQRERRDKYSPDGHNRGSSPGPKPEPVGSPVRDGRGSSE
>Solyc03g082380.4.1
MSGRFSRSIYVGNLPADIKELEVEDLFYKYGRILDIELKIP-
PRPPCYCFVEFESSRDAEDAIRGRDGYNFDGCRLRVELAHGGRGPSSSDRRGSYGSGGGGGGGRHGISRHSDYRVIIRGLPSSASW
QDLKDHMRKAGDVCFAEVSRDSEGTFGLVDYTNYEDMKYAIRKLDDTEFRN-
PWTRTYIRVREYKGSPSRSRSRSRSRSRSRSRSRRSPARSISRSPPPKSRSASPVKSTRSRSLSRSMSRSRSRSRSRSRSKSRSPSRSRSASPQ
QARSNSG
>Solyc04g074040.3.1
MSHFGRSGPPDIKDTYSLLVLNVTFRTTADDLFPLFDKYGKVVDVFIPRDRRTGDSRG-
FAFVRYKYQDEAQKAVEKLDGRVVDGREIMVRFAKYGPNAERIDKGRILEPVQRPKGRSRSRSPRPRHRDHRDKDSRRRSRSRSRS
RSRGRYERDQYRGRDRDNRHRSRSRSPDYHRGRGRGKYDEDRRSRSRSHGRSAS-
PARRSPSPRRSPSPRRTTPPRDASPDGRNHKDRSPTPKSISPRGRRAGSRSPLPRSDADD
>Solyc05g054920.5.1 SlySF4N
MPRYDDRYGGTRLYVGHLSSRTRSRDLEDVFSRYGRVRDVDMKRDYAFVEFSDPRDADDARY-
GLNGRDVDGSRVIVEFAKGVPRGPGGSREFGGRGPPPGTGRCFNCGIDGHWARDCKAGDWKNKCYRCGDRGHIERNCQNSPK
KLKRDRSYSRSPSPRRGRSRSRSRSYSRGRSYSRSRSPVKRDRSI-
EREEKRSRSPRHHRSSPPPSKGRKHSLSPDERSPVERGTPSPRDDRATNGSDRSRSPKDDVRMDERGDISPVEENGRSRSNSPIHRED
RSPVEDGSPTGDYENHGSPRGSPRGSESP
>Solyc06g009060.4.1
MVGVVTHRQIVIIVAIIVAIMVVVIITNLERPNVPSIEALLVTGLPH-
SASWQDLKDHMRRAGDVCFSQVFREGSGTTGIVDYTNYDDMKYAIKKLDESEFRNAFSRSTIRVKEHDSRSRSRSRSYSRGKSGSRS
RSRSYSRSRSRSKSPKAKSSKRTRSRSRSVSS-
QPRSGLKGRSLSRSPSRSRSPVPSRPKRVSKSPKPRDSRRSQSLSKSPKPRDSRRSESPSKSPKLRDSRRSKSMSKSPEPRNSRRSPSRSKS
PKPRNSRRSPSRSRSRSRSGSLSR
>Solyc09g005980.4.1
MPRYDDRVGNSTRLYVGHLSSRTRSRDLERAFSKYGRVRDVDMKHDYAFVEFSDPRDAD-
DARYYLDGRDIDGRRIIVEFAKGVPRGPGGSREYLGKGPAPGSGRCFNCGLEGHWARDCKAGDWKNKCYRCGERGHIERKCPN
SPKKLSRRSYSRSPARSKSRSRSRSRSPRRSYSRSRSYSQSRSPPPKREQVDQVKRSRSYSRSPEPRKDSPSPPPKTRKRSPTPEEGSPMEA
KSPSSPMREEGAYSQSPRERSVSPSSTRRDSPAPRKYDDDSPAEANGGSRSPSPKYQRNHEDDEDEGEFRNQRSGRESQSP
>Solyc09g075090.1.1
MRNMLFASLYPARLHFLKTLIFKISSSSTLSALDLPSRSSRTLYVGNLPGDVREREVED-
LFYKYGPIAHIELKIPPRPPGYAFVEFEEARDAEDAIRGRDGYDFDGHRLRVELAHGGRGNSSSNDRYGGGGGGRGQRGGGVSRR
SDYRVLVTGLPHSASWQDLKDHMRRAGDVCFSQVFRDGSGTTGIIDYTNYDDMKYAIK-
KLDDSEFRNAFSRATIRVKEYDRSRSRSRSRSRSYSRGKSVSRSRSRSRSRSRSKSKSKSPKVKSSKRSRSRSRSVSSQSRSGSKGRPVSRF
WICAAVCGFAY
>Solyc10g009330.3.1
MPTTTNKGYAFVYFEDDRDAADAIRGTDNMPFGYERRRLSVEWAKGERGRHHDGGPKSGG-
NQRPTKTLFVINFDPIRTRVRDIEKHFEPHGKVLHVRIRRNFAFVQFENQEEATRALECTHMSKVLDRVVSVEYALKDDDERGDKY
NSPRRDYGRQRDSPYRRSPSPVYRRNRPSPDYGRPRSPVHNGPSYDRYRSPQYGRYRSRSPVRRS
>Solyc11g072340.2.1
MRPIFCGNVEYNARQSELERLFRRYGRVDRVDMKSGFAFVYMDDERDAEDAIRGLD-
RIEFGRKGRRLRIEWSKEERNGRRPETSRKSSSSVKPSKTLFVINFDPYSTRSRDIERHFDPYGKILNIRIRRNFAFVQYETQEDATRAL
DATNMSKLMDQVITVEYANKDDDDRRNGFSPDRNRDRGLKRGYDRGRSRSPYGRERGSPDY-
GRGRARSPSPIRQGRSSPDYGRRPSPNPNHRERDSEYGSGRSPNMRKERNPDHGNGHSPNPRRLRAGSENGEVHSPPEEGLLESGP
SPPRVGRRGKYSPDDYRGRSRSPSPRSKPEEIGSPRYGAAESPLPERHRSLSPPTRERSRS
>Nta00551.t1
MRPIFVGNFEYDTRQSELERFFSKYGRIERVDMKSGFAFVYFEDERDAADAIR-
GTDNMPFGYERRCLSVEWAKGERGRHRDGPKSGGNQRPTKTLFVINFDPTCTRTRDIERHFEPYGNVLHVRIRRNFAFVQFENQE
DATRALECTHMSKILDRVVSVEYALKDDDERGDRYDSPRRDYGRQRD-
SPYRRSPSPMYRRGRPSPDYGRPHSPVRNGPSYDRYNSPDYGRYRSRSPVRRSRT
>Nta01034.t1

MVNYFQLLFLNYWSKNWPATLFWQSQKPEYFFDRALAIFASKGFRCVSKMSRVYVGN-
LDPRVSERELEDEFRIFGVIRSVWVARRPPGYAFIDFDDKRDAQDAIRELDGKNGWRVELSHNSREGRGGRGGGRGRSGGSDLKC
YECGEPGHFARECRLSGAPGRRRSRSPPRYRRSPSYGRRSYSPHGRSPQRRSLSPRGRSYSRSPYRGQDKVPYANGNGLRERRRSRS
>Nta03609.t1
MRGRSCSYSPSPHRGYSKRCRSPSPRGRFGGRLSDPPTSLLVRNLHHDCRREDLRGPFGEFG-
PIKDIYLPRDYYTGEQRGFGFVQYLDPADAADAKYEMDGQVLLGREITVVFAEENRKKPSEMRARERSRGRGSSQLSYSRSPRYRRS
YSRSPRYARAYSRSPDYYSLPPRRRYYSRERSYSRSPYGSRSRSPVWSRSRSRSRSFDDKSSLAVLRL
>Nta04206.t1
MQLLIKNLTIVSKRYFHSASALRLLLQLGFSILQNGRMRPIFCGNFEFD-
TRQSELERLFKRYGKVDRVDMKSGFAFVYMDDERDAEDAIQGLDRIDFGRKGRRLRVEWSKEERSRKPEVSRKSSSSFRVSKTLFVI
NFDPYNTRTKDLERHFDPHGKILNIRIRRNFAFIQFESQEDATRAL-
DATNMSKLMDRVITVEYAIKDDDDRRDGSGPGKTHDRSPRRGYDRGRSRSPYGRDRPSPDYGRGRDRPSPDYGRGRDRLSPDYGR
GRNRLSPDYGRGRDRLNPDYGRGPSPSPKHRERNSENARGHSPAVGKERN-
PGHGNGRTPSPRRERAGPGNGLVSSPMESRSPGYGDRPSPSPQRERRKKYSPDGYNPGSSPSSKPGPVESPVRDGRESSERYRSRSPP
LRERSRS
>Nta04569.t2
MVLQSYSLLRPLPFAFLMTLLSPHYLTCFNIDSMIISDPVAAVEFLPTDFSFPFPP-
SPPHSWGVLGGFAFVYMEDERDADDAIRRLDRIEFGKKGRRLRVEWTKEDRGGSRRPDSSRKPAANTKPSKTLFVINFDPVHTRTR
DIERYFEPYGRISNVRIRRNFAFVQYESEDDASRALEATNMSKFMDRVIS-
VEFAIRDDDDRRNGRSPDRRGRDMSPGRRGYVRRRSPSPYRRDRGSPDYGHGALLNSRPRTRGSPEYRRAESPVNERYHSRSPPPR
EVSILRRKLPSKISSPRKEKEKENLPNKKLV
>Nta04907.t1
MQGLSTSLLSSGLHILADVVLYLIPKGDYNFDLTDVHSAYEIFRLSCHAFLWPYNGVFTTT-
WFAFVYFEDERDAADAIRDLDDIPFGYDKRWLSVEWAKGECGRPHDGSKVAANQRPTKNLFVIKFDPNRTRLRDIERHFELYGE
VLNVRIRRTFAFVQFENQEDATKALESTHMSKILNRVVSVEYALRDDDKRGDRYG-
SPRRDYGRHGDSPYRRSPSPMYQRSRPSPDYGRSHSPAFDKYNGLSYDRYRSPEYGRYHSRSPIRRSRT
>Nta08283.t1
MSHFGRTGPPDITDTYSLLVLNITFRTTADDLYPLFDKYGKVVDIFIPRDRRTGESRG-
FAFVRYKYADEAQKAVDRLDGRVVDGREMAVQFAKYGPNAERIQQGRIIEKVSRIKGSSRSRSPRRRYRDDHYRDREYRRSRSRSV
DRYERDRYKRRERDYRRRSRSRSLSPDYDRDRGRGRDKKHHRRSPFDSAS-
PPRRSPSPYRKESPRGSLSPKKESPEKRSHIERSPTPRSRSPPGRAMDSRSPSPRADEE
>Nta08815.t1
MGRLSRTIYVGNLPGDIREREVEDLFYKYGHIVEIELKVPPRPPGYAFVEFEDSRDADDAIR-
GRDGYDFDGHRLRVELAHGGQGSSSYDRHSSYSSGSRGGVSRRSDHRVLISGLPSSASWQDLKDHMRRAGDVCFSQVFRGRDGM
RGIVDYTNYEDMKYAIRK-
LDDSLFRNQFSRAYIRVEEYDKRRSYSRSPSPYYSRGRVYSRSRSPRRSYSSQSRSVSPRGKYSRRSASVSRSRSFSPARSVSRSQSRSRSP
LSPPHRRRHARSPSRSRSRSFSYSRSSGISD
>Nta09511.t1
MKSGFAFIYMEDERDADDAIRRLDRIEFGKKGRRLRVEWTKEDRGGSRRPESSRK-
PAANTKPCKTLFVINFDPVHTRTRDIERYFEPYGRISNVRIRRNFAFVQYESEDDASRALEATNMSKFMDRVISVEFAIRDDDDKRN
GRSPDRRGRDMSPGRRGYDRRRSPSPYRRDRGSPDYGHGALLNSRPRTRGSPEYRRAESPVNERYHSRSPPPRERSRS
>Nta10646.t1
MGRLSRTIYVGNLPGDIRESEVEDLFYKYGPIVEIDLKIPPRPPGYAFVEFEDPRDADDAIRGRDGYKFDGHRLRVSCLSFASISPWS
>Nta12053.t2
MSRSSRTLYVGNLPGDIREREVEDLFYKYGPIAHIELKIPPRPPGYAFVEFEEVRDAEDAIR-
GRDGYDFDGHRLRVELAHGGRGQSSNDRYGGGDGGGGRGHRGGVSRRSDYRVKITGLPHSASWQDLKDHMRRAGDVCFSQVF
RERRGTTGIVDYTNYNDMKYAIK-
KLDDSEFRNAFSRSTIRVKEYDHSRSQSRSRSRSRSYSRGKSGSRSRSRSRSVGRSRSKSKSPKAKSSRRSRSRSRSVSSRSRSGSKGRSIS
RSPSRSRSPAPSRPKRLSKSPKRRSPSQSQSRSRSRSRSRGLSS
>Nta13274.t1
MGRHRSRSRSYSPVRRKRYDEPRDRRH-
DRRSPAPSGLLVRNISLSARPEDVRVPFERFGPVKDVYLPKNYYTGEPRGFGFVKFRYAEDAAEAKAHLNCTVIGGREISIVFAEEN
RKTPQEMNRVLRTSGPSARGSYRRHSPQRFPSRRYHSYSRSAS-
PARRDSRDRDRENRDDYYSPRRCRSKSPSVSPRDERNYRLNGRSSRQSRQMSRSNTPRIQKKGRPSPSPRDNRLIVHDLDSARRRLE
RSGRNVDSSARSRSRSYSPH

>Nta16341.t1
MSRVYVGNLDPRVTERELEDEFRVFGVIRSVWVARRPPGYAFIDFDDQRDARDAIRE-
IDGKNGWRVELSHNSRGGGGGGRGGGRGRSGGSDLKCYECGEAGHFARECRGRGAPGKRRSRSPPRYRRSPSYGRRSYSPRGRSP
RRRSPSPRGRSYSRSPYRGRDEVPYANGNGLRDRIRSRS
>Nta17668.t1
MRRRSYSPSPPRGYGRRGGRSPSPRGRYGGRSRDAPTSLLVRNLRHD-
CRPEDLRRPFGQFGPVKDIYLPRDYYTGQPRGFGFVQFVDPADAAEAKYQMDGQGFQGRQLTVVFAEENRKKPTEMRARERSGS
GRSRSYDRRRYSPQYSRSPPPRYARSRSRGYSPKRRQYSRSVSPEEKRYSRER-
SYSRSPARDISPPYNGSRSRSQTPVREHSPYDDGRRSRSRSPVKERSPVRGHSRSPSRSRSPGDVRYSRDPDHDVSPRH
>Nta18639.t1
MRPIFCGNVEYNARQSELERLFRRYGKVDRVDMKSGFAFVYMDDERDAEDAIRGLD-
RIEFGRKGRRLRIEWSKEERSSRRPESSRKSSSSVKPSKTLFVINFDPNNTRSRDIERHFDPYGKILNIRIRRNFAFVQYETQEDASRAL
DATHMSKLMDQVITVEYANKDDDDRRTGFSPDRNRDRGGLRRGYDRDRSRSPYGRERGSPDY-
GRGRARSPSPLRHGRSSPDYGRGASPNPNHRERNSEYGRGHSPSMRKERNPDQGNGHSPNPRRLRAGSENGHVSSPPEEGMLDD
RRASPSPPRGRREKQSPDGYRGRSPRSKPEEIDSPGYAAAESPLPERHRSDSPPARERSRS
>Nta21382.t1
MSRSSRTLYVGNLPGDIREREVEDLFYKYGPIAHIELKIPPRPPGYAFVEFEEVRDAEDAIR-
GRDGYDFDGHRLRVELAHGGRGQSSNDRYGGGGGGGRGQRGGVSRRSDYRVKITGLPHSASWQDLKDHMRRAGDVCFSQVFR
ERRGTTGIVDYTNYDDMKYAIK-
KLDDSEFRNAFSRSTIRVKEYDHSRSQSRSRSRSRSYSRGKSGSRSRSRSRSVSRSRSKSKSPKVKSSRRSRSRSRSVSSRSRSGSKGHFISR
SPSRSRSPAPSRPKHLSKSPKRRSPSQSQSRSRSLSRSRSRGLSS
>Nta21547.t1
MSRSSRTIYVGNLPGDIREREVEDLFYKYGPIAHIDLKVPPRPPGYAFVEFEEARDADDAIR-
GRDGYDFDGHRLRVELAHGGRGNSSANDRYSGNSSGRNHKFGAPKRTEYRVLVTGLPHSASWQDLKDHMRRAGDVCFSQVFR
EGGGTTGIVDYTNYNDMKYAIK-
KLDESEFRNAFSRSTIRVKEHNSRSRSRSRSYSGRKSGSRSRSPSRSRSRSKSPKAKSSKRTRSRSRSVSSQPHSGLKGRSVSRSPSRSRSP
VPSRPKHASKSPTPHNSRKSRSVSKSPKPRNSRRSQSMSKSPKPRSSRRSQSRSRSRSGSLSR
>Nta21928.t1
MRGRSYSPSPPRGYGRRGRSPSPRGRYAGRSRDDPTTLLVRNLRHDCRSED-
LKKPFGQFGPVKDIYLPRDYYTREPRGFGFIQYLDPADAGEAKYQMDGQVFQGRQLTVVFAEENRKKPQEMRARERGSGRGGRN
YDRRGTPPRFHNSPRYSRSPPPRSRDYYSPPKRRQYSRSVSPEEKRYSRER-
SYSPRAGQGRSYSQSPPREQSPPYNGSRSRSQSPVKEEQSPPYNGSRSRSRSPVRARSPVRGPSRRRSPSQSRSRSPDAVHYSRNPDRD
VSPRH
>Nta23360.t1
MSGRFSRTIYVGNLPADIKEWEVEDVFYKYGRILDIELKIP-
PRPPCYCFVEFENARDAEDAIRGRDGYNFDGCRLRVELAHGGRGPPSSSDRRGGYGRSDGGGRHGISRHSEYRVIVRGLPSSASWQ
DLKDHMRRAGDVCFAEVSRDSEGTFGLVDYTNCDDMKYAIRK-
LDDTEFKNPWTRTYIRVREHKRSPSRSRSRSRSRSRSPRGSRSPKRSRSKSAGRSVLRSPPAKSRSASPVKSTRPRSRSKSRSRSASPRQA
RSNSG
>Nta23756.t1
MGAQQKHLHVTCKHASSFLLQKAKKNSLELSIQRH-
SPPTKQKEKKVYLSKAHQYCEKPLIELTITLDSLPERAMSRVYVGNLDPRVTERDLEDEFRIYGVLRSVWVARQPPGYAFIEFDDRR
DALDAIRELDGKNGWRVELSHNSSGRGGGRGGRGGDDTKCYECGEPGHFARECRLCIGS-
RGLGSGRRRSPSPQYRRSPSYGRRSYSPHGKSPRGRSLSPRRGRSYSKSPPNHRGRYVSPYADGLSPCRGRGSYSRSPAYRRHRDSPY
ASGA
>Nta28584.t1
MSHFGRTGPPDIKDTFSLLVLNVTFRTTADDLFPLFDKYGKVVDVFIPRDRRTGDSRG-
FAFVRYKYQDEAQKAVEKLDGRVVDGREIMVRFAKYGPNAERIDKGRILEPVNRTGGRSRSRSPRSRHRDHYRDKDSKRRSRSRS
GGRYDRDRYRGRDKDYRQRSRSRSRSPDYHKARGRGKYDEERRSRSRSHGSAS-
PVRRSPSPRRSPSPRRTTPSRDASPDGRNRKERSPTPKSVSPRGRRAGSRSPLPCSDADD
>Nta28585.t1
MSHFGRTGPPDIKDTFSLLVLNVTFRTTADDLFPLFDKYGKVVDVFIPRDRRTGDSRG-
FAFVRYKYQDEAQKAVEKLDGRVVDGREIMVQFAKYGPNAERIDKGRILEPVQNTRGRSRSHSPRSRHRDHYRDKDSKRRSRSRS
RDRYDRDRYRGRDKDYRRRSRSRSPDYRK-
DRERGKYDEERRSRSRSHRSVSPARRSLSPRRSPSPRRTTPSRDASPDGRNHKDRSPTPKSVSPRGRRGGSRSPLPHSDADD

>Nta29553.t1 NtaSF4Nb
MPRYDDRYGGTRLYVGHLSSRTRSRDLEDVFSRYGRVRDVDMKRDYAFVEFSDPRDADDARY-
GLNGRDVDGSRITVEFAKGVPRGPGGSREFGGRGPPPGTGRCFNCGLDGHWARDCKAGDWKNKCYRCGERGHIERNCQNSPK
KLKRGRSYSRSPSPRRGRSRSRSYSRGRSYSRSRSPVRRDSRSPVKRDQSIEHEERRSSSPRPRRSSPPPSKGRKYSPSPDERSPQERGTPS
PKGDRAANGSEYSRSPTDDAGIDERRNLSPIEENGRSHSNSPIHRENGSPMGDDENHGSPRGSESP
>Nta33006.t1
MSRSSRTIYVGNLPGDIREREVEDLFYKYGPIAHIDLKVPPRPPGYAFVEFEEARDADDAIR-
GRDGYDFDGHRLRVELAHGGRGNSSANDRYSGNSSGRNHKFGAPKRTEYRVLVTGLPHSASWQDLKDHMRRAGDVCFSQVFR
EGGGTTGIVDYTNREDMKYAIK-
KLDESEFRNAFSRSTIRVKEHNSRSRSRSRSYSGRKSGSRSRSASRSQSRSKSPKAKSSKRTRSRSRSVSSQPHSGLKGRSVSRSPSRSRSP
VPSRPKHVSKSPPPRDSRRSRSVSKSPKPRNSRRSQSMSKSPKPRISRRSQSRSRSRSRSGSLSR
>Nta34446.t1
MSSLGGGDDMSSASDLPFLGPRSTMDLQEFSRKPRHNVSVTLGELLKRVGDSTEETYNNNS-
LELGNHYSNIASPSSFPFILSFHNLSYSVKVKTKMALPKFLRRGNKDNELLDDNMKMLLNDISGEAREGEIMAVLGASGSGKSTLID
ALADRISRESLKGTVTLNGEVLESKLLKVISAYVMQDDLLFPMLTVEETLMFSAEFRL-
PGTLSKSKKKARVQALIDQLGLTTAAKTVIGDEGHRGVSGGERRRVSIGIDIIHDPIVLFLDEPTSGLDSTSAYMVVKVLQRIAQSGG
IVIMSIHQPSYRILSLLDHLIILSRGNTVFTSSPSSLQQFFAEFGNPIPENENRIEFALD-
FIRELEGTPNGTKNLVEFNKTWQRKKTSNSSSLFYNGTKPSLKDAISASVSRGKLVSGATNIDSSLSSSNIPKFANPFWVDMVVIAKR
SMLNSMRMPELFGMRFGAVVVTGIILATIFWKLDNSPKGVQERVGFFAFAM-
STTFYTCAEAIPVFLQERYIFMRETAYNAYRRSSYVLSHAIISLPSILVLSIAFAVTTYWSVGLAGGVSGFLFFLLFMVASFWAGSSFVT
FLSGVIYNVMMAYTVVVAILAYFVLFSGYFISRDRIPPY-
WIWFHYMSLVKYPYQGVLQNEFDDPLKCFVKGIQIFDGSPLRAVPEQLKIKLLHNMSKTLGMNITSSTCLTTGADILKQTGVNDL
NKWSCLWITIALGFFFRILFYFALVIGSKNKRRVSSFYKQGFKKQPAVLVTNFALKFENRAV-
KMPRYDDRSGNSTRLYVGHLSSRTRSRDLERVFSKYGRVRDVDMKHDYAFVEFSDPRDADDARYYLDGRDVDGRRIIVEFAKGVP
RGPGGSREYLGKGPAPGSGRCFNCGLEGHWARDCKAGDWKNKCYR-
CGERGHIEKKCPNSPKKLSRRDRSYSRSPVRSRSHSRSRSPRRRSYSRSRSYSPSRSPAPRRGKVDIERRSRSYSRSPEPKKASPSPSLSPP
PKTRKHSPTPEDGSPTEAMRTTTREEGSYSQSPRERSASPLSPRRGDSPAPERDED-
SPAEANGGSRSPSPNYQRNRADDDEDEGEYNNQRSGRESESP
>Nta34589.t3
MSGRFSRTIYVGNLPADIKEWEVEDLFYKYGRILDVELKIP-
PRPPCYCFVEFENARDAEDAIRGRDGYNFDGCRLRVELAHGGRGPPSSSDRRGGYGRSDGGGRHGISRHSEYRVIVRGLPSSASWQ
DLKDHMRKAGDVCFSEVSRDSEGTFGLVDYTHYDDMKYAIRK-
LDDTEFKNPWTRTYIRVREHKRSPSRSRSRSRSRSPRGSRSRSRSRSRIPKRRSKSAGRSVLRSPPAKSRSASPVKSTRPRSRSKSKSKSR
LRSASPRQTIVDGYLNRRFCSCLNHSDVS
>Nta35550.t1
MGRHRSRSRSYSPVRRKRHDEPRDRRH-
DRRSPAPSGLLVRNISLSARPEDVRVPFERFGPVKDVYLPKNYYTGEPRGFGFVKFRYAEDAAEAKAHLNCTVIGGREIRIVFAEEN
RKTPQEMNRVLRTSGPSARGSYRRHSPQRFPSRRYHSYSRSAS-
PARRDSRDRDRENRDDYYSPRRCRSKSPSVSLRDERNYRLNGRSSRQSRQMSRSNSPRIQKKGRSSPSPRDNSPIAHDVDSAPRRLE
RPGRNADSSARSRSRSYSPR
>Nta38506.t1
MRPIFCGNFEFDTRQSELERLFKRYGKVDRVDMKSGFAFVYMDDERDAEDAIQGLDRID-
FGRKGRRLRVEWSKEERSRKPEVSRKFSSFRVSKTLFVINFDPYNTRTRDLERHFDPHGKILNIRIRRNFAFIQFESQEDATRALDAT
NMSKLMDRVITVEYAIKDDDDKRDGSGPGKTHDRSPRRGYDRGRSRSPYGRDRPSPDY-
GRGRDRPSPDYGRGRDRLSPDYGRGRNQLSPDYSRGRDRLNPDYGRGPSPSPKHRERNSENARGHSPAVGKERNPGHGNGRTPS
PRRGRSGPGNGLVSSPLESRSPGYGDRPSPSPQRERRKKYSPDGYNPGSSPSSKPEPVESPVCDGRESSERYRSRSPPLRERSRS
>Nta38865.t1
MSGRFSRTIYVGNLPADIKESEVEDLFYKYGRILDIELKIP-
PRPPCFSFVEFESSRDAEDAIRGRDGYNFDGCRLRVELAHGGRGPSSSSDRRGSYGSSGGGGRYGVSRHSDYRVIVRGLPSSASWQD
LKDHMRKAGEVCFAEVSRDSEGTFGMVDYTTYEDMKYAIRKLDDTEFRNPWTRTYIRVKEY-
KRSPSRSRSRSRSPKRSRSRSRSRSPKRSRSKSPGRSLSRSPSPKSRSASPVKPTRSRSMSRSRSRSISRSLSRSRSASPRQARSNSG
>Nta42500.t1
MSHFGRTGPPDIKDTFSLLVLNVTFRTTADDLFPLFDKYGKVVDVFIPRDRRTGDSRG-
FAFVRYKYQDEAQKAVEKLDGRVVDGREIMVRFAKYGPNAERIDKGRILEPVHRTGGRSRSRSPRSRHRDHYRDKDSKRRSRSRS

GGRYDRDRYRGRDKDYRQRSRSRSPDYHKARGRGKYDEERRSLSRSHGSAS-
PVRRSPSPRRSPSPRRTTPSRDASPDGRNRKERSPTPKSVSPRGRRAVSRSPLPRSDADD
>Nta45559.t1
MRPIFCGNVEYNARQSELERLFRRYGKVDRVDMKSGFAFVYMDDERDAEDAIRGLD-
RIEFGRKGRRLRIEWSKEERSSRRPESSRKSSSSVKPSKTLFVINFDPHNTRSRDIERHFDPYGKILNIRIRRNFAFVQYETQEDASRAL
DATHMSKLMDQVITVEYANKDDDDRRTGFSPDRNRDRGGLRRGYDRDRSRSPYGRERGSPDY-
GRGRVRSPSPLRHGRSSPDYGRGASPNPNHRERNSEYGRGHSPSMRKERNPDQGNGHSPNPRRLRAGSENGHVSSPPEEGMLDD
RRASPSPPRGRREKQSPDGYRGRSPRSKPEEIDSPGYAAAESPLPERHRSDSPPARERSRS
>Nta49739.t1 NtaSF4Na
MPRYDDRYGGTRLYVGHLSSRTRSRDLEDVFSRYGRVRDVDMKRDYAFVEFSDPRDADDARY-
GLNGRDVDGSRITVEFAKGVPRGPGGSREFGGRGPPPGTGRCFNCGLDGHWARDCKAGDWKNKCYRCGERGHIERNCQNSPK
KLKRGRSYSRSPSPRRGRSRSHSYSRGRSYSRSRSPVRRDSRSPVKS-
DSRSPVKRDRSIEHEERRSRSPRPHRSSPPPSKGRKYSPSPDERSPQERGTPSPRGDRAANGSDHSRSPTDDAGIDERRNLSPIEENGR
SRSNSPIHRDNGSPVENGSPMGDDENHASPRGSESP
>Nta50235.t1
VWVARRPPGYAFIEFDDRRDALDAIHELDGKNGWRVELSHNSSGRGGGRGGRGGDDTKCYE-
CGEPGHFARECRLRIGSRGLGSGRRRSPSPRYRRSPSYGRRSYSPLGKSPRGRSLSPRRGRSYSRSPPNRRGRYVSPYANGLSPRRGRG
SYSRSPAYRRHRDSPYAKGA
>Nta52147.t1
MPRYDDRSGNSTRLYVGHLSSRTRSRDLERVFSKYGRVRDVDMKHDYAFVEFSDPRDAD-
DARYYLDGRDVDGRRIIVEFAKGVPRGPGGSREYLGKGPAPGSGRCFNCGLEGHWARDCKAGDWKNKCYRCGERGHIEKKCPN
SPKKLSRRDRSYSRSPVRSRSRSRSPRRRSYSRSRSYSPSRSPP-
PRRGKADLEKRSRSYSRSPEPRKESPSPSPSPPPKTRKHSPTPEEGSPMEAMRTTTGEEGSYSQSPRERSASPLSPRRGDSPAPERDEDS
PAEANGGSRSLSPKYQRNRADDDEDEGEYNNQRSGRESESP
>Nta57040.t1
MRPIFVGNFEYDTRQSELERFFSKYGRIERVDMKSGFAFVYFEDERDAADAIR-
GTDNMPFGYERRRLSVEWAKGERGRHRDGPKSGGNQRPTKTLFVINFDPTCTRTRDIERHFEPYGNVLHVRIRRNFAFVQFDNQE
DATRALECTHMSKILDRVVSVEYALKDDDERGDRYNSPRRDYGRQRD-
SPYRRSPSPMYRRGRPSPDYGRPHSPVRNGPSYDRYNNSPDYGRYRSRSPVRRSRT
>Nta57701.t1
MKIHIFFRNLASSTTEYSKSVTFFIRIFLSNSKGFRCVKKMSRVYVGNLDPRVSERELEDE-
FRIFGVIRSVWVARRPPGYAFIDFDDLRDAQDAIRELDGKTGWRVELSHNSREGRGGRGGGRGRSGGSDLKCYECGEPGHFAREC
RSRGAPGRHRSRSPPRYRRSPSYGRRSYSPRGHSPRRRSVSLRGRSYSRSPYRGRDEVPYANGNGLRERRRSRS
>Nta61369.t1
MSHFGRTGPPDITDTYSLLVLNITFRTTADDLYPLFDKYGKVVDIFIPRDRRTGESRG-
FAFVRYKYADEAQKAVDRLDGRVVDGREMAVQFAKYGPNAERIQQGRIIEKVSRIKGSSRSRSPRRRYRDDHYRDREYRSRSRSVD
RYERDRYKRRERDYRRRSRSHSLSPDYDRGRGRGRDTKHHRRSPFDSAS-
PPRRSLSPYRKESPRRSLSPKKRSPEKRSHIERSPTPRSRSPLGRAMDSRSPSPHADEE
>Nta61868.t1
MFEQAAFFWLMVSGIMGRLSRTIYVGNLPGDIREREVEDLFYKYGHIVEIDLKVP-
PRPPGYAFVEFEDSRDADDAIHGRDGYDFDGHRLRVELAHGGQGSSSYDRHSSYSSGSRGGVSRHSDHRVLISGLPSSASWQDLK
DHMRRAGDVCFSQVFRDRDGLRGIVDYTNYEDMKYAIRK-
LDDSLFRNQFSRAYIRVEEYDKRLHIIQEAEVTQEAGVLDGAIAARAEGLVSDCLFHHLQKI
>Nta63212.t1
MSGRFSRTIYVGNLPADIKEWEVEDLFYKYGRILDIELKIP-
PRPPCFSFVEFESSRDAEDAIRGRDGYNFDGCRLRVELAHGGRGPSSSSDRRGSYGSSGGGGRYGVSRHSDYRVIVRGLPSSASWQD
LKDHMRKAGDVCFAEVSRDSEGTFGMVDYTNYEDMKYAIRKLDDTEFRNPWTRTYIRVREY-
KRSPSRSRSRSRSPRRSRSKSPGRSLSRSPTPKSRSASPVKPTRSRSISRSRSRSISRSLSRSRSASPRQARSNSG
>Nta63630.t1
EPRGFGFVQYLDPADAADAKYEMDGQVLLGREITVVFAEENRKKPSEMRAREHSRGRGSSQL-
SYSRSPRYRRSYSRSPRYARTYSRSPDYYSPPPRRRYYS
>Nta67806.t1
MGFSLIGYYSEMSRVYVGN-
LDPRVTERELEDEFRVFGVIRSVWVARRPPGYAFIDFDDQRDARDAIREIDGKNGWRVELSHNSRGGGGGGGGRGGGRGRSGGS

DLKCYECGEAGHFARECRGRGVPGKRRSRSPPRYRRSPSYGRRSYSPRGR-
SPRRRSPSPRGRSYSRSPYRGRDEVPYSNGNGLRDRIRSRS
>Nta70457.t1
MRRRSYSPSPPRGYSRRGGRSPSPRGRYGGRSRDAPTSLLVRNLRHD-
CRPEDLRRPFGQFGPVKDIYLPRDYYTGQPRGFGFVQFVDPADAAEAKYQMDGQGFQGRQLTVVFAEENRKKPTEMRARERSGS
GRSRSYDRRRYSPQYSRSPPPRYARSRSRSRGYIPKRRQYSRSVSPEEKRYNRERSYSH-
SPAGDISPPYNGSRSRSQTPVREHSPYDDGRRSRSRSPVKERSPVRGRSRSPSRSRSPDDARYSRDPDHDVSPQH
>Nta73120.t1
MRGRSYSPSPPRGYGRRGRSPSPRGRYAGRSRDDPTTLLVRNLRHDCRSED-
LKKPFGQFGPVKDIYLPRDYYTREPRGFGFIQYLDPADAGEAKYQMDGQVFQGRQLTVVFAEENRKKPQEMRARERGSGRGGRN
YDRRGTPPRFHNSPRYSRSPPPRSRDYYSPPKRRQYSRSVSPEEKRYSRER-
SYSPRAGQGRSYSQSPPREQSPPYNGSRSRSQSPVKEEQSPPYNGSRSRSRSPVRARSPVRGPSRRRSPSQSRSRSPDAVHYSRNPDRD
VSPRH
